# Genome-Wide Association Mapping Reveals Novel Putative Gene Candidates Governing Reproductive Stage Heat Stress Tolerance in Rice

**DOI:** 10.3389/fgene.2022.876522

**Published:** 2022-05-10

**Authors:** K. T. Ravikiran, S. Gopala Krishnan, K. P. Abhijith, H. Bollinedi, M. Nagarajan, K. K. Vinod, P. K. Bhowmick, Madan Pal, R. K. Ellur, A. K. Singh

**Affiliations:** ^1^ Division of Genetics, ICAR-Indian Agricultural Research Institute, New Delhi, India; ^2^ Rice Breeding and Genetics Research Centre, ICAR-IARI, Aduthurai, India; ^3^ Division of Plant Physiology, ICAR-Indian Agricultural Research Institute, New Delhi, India

**Keywords:** rice, GWAS, marker-trait association, quantitative trait nucleotides, reproductive stage heat stress tolerance

## Abstract

Temperature rise predicted for the future will severely affect rice productivity because the crop is highly sensitive to heat stress at the reproductive stage. Breeding tolerant varieties is an economically viable option to combat heat stress, for which the knowledge of target genomic regions associated with the reproductive stage heat stress tolerance (RSHT) is essential. A set of 192 rice genotypes of diverse origins were evaluated under natural field conditions through staggered sowings for RSHT using two surrogate traits, spikelet fertility and grain yield, which showed significant reduction under heat stress. These genotypes were genotyped using a 50 k SNP array, and the association analysis identified 10 quantitative trait nucleotides (QTNs) for grain yield, of which one QTN (*qHTGY8.1*) was consistent across the different models used. Only two out of 10 MTAs coincided with the previously reported QTLs, making the remaing eight novel. A total of 22 QTNs were observed for spikelet fertility, among which *qHTSF5.1* was consistently found across three models. Of the QTNs identified, seven coincided with previous reports, while the remaining QTNs were new. The genes near the QTNs were found associated with the protein–protein interaction, protein ubiquitination, stress signal transduction, and so forth, qualifying them to be putative for RSHT. An *in silico* expression analysis revealed the predominant expression of genes identified for spikelet fertility in reproductive organs. Further validation of the biological relevance of QTNs in conferring heat stress tolerance will enable their utilization in improving the reproductive stage heat stress tolerance in rice.

## Introduction

Climate change and global warming are seriously affecting agricultural productivity. The mean surface temperature of the earth today is 0.8°C higher than that in the pre-industrial era and is projected to increase up to 4.8°C by the end of this century (IPCC, 2014). In India also, there has been a concurrent increase of 0.63°C since 1986, triggering intermittent heatwaves, and this is predicted to increase to 4.7°C by the end of 2100. The heatwave episodes are expected to intensify particularly in the Indo-Gangetic plains of India, where rice–wheat is the most prevalent cropping system (Im et al., 2017; Krishnan et al., 2020). Rice is the major staple food crop of India with an area of 43.79 million hectares (mha) and a production of 116.42 million tons (mt) during 2018–19 (GoI, 2019). It is highly sensitive to heat stress at the reproductive stage with the optimum temperature ranging from 22 to 28°C (Prasad et al., 2006). Temperatures beyond the tolerant threshold (35°C) at anthesis and booting will adversely affect rice yields (Satake and Yoshida, 1978; Yoshida et al., 1981). Simulation models have predicted that for every 1°C rise in ambient temperature during the sensitive stages, the rice yield will suffer by 2.5 up to 10% (Baker et al., 1992; Peng et al., 2004). The temperatures of many tropical rice-growing countries have already reached ∼33°C, and any further increase will have severe taxing on the grain yield and quality (Wassmann et al., 2009). For instance, a heatwave (∼36°C) for 2 consecutive days in early April 2021 coupled with low rainfall and humidity has devastated the 68,000 acres of a spring-grown rice crop of Bangladesh with an estimated economic loss of $39 million (Hossain, 2021).

Heat stress is one of the complex abiotic stresses, to which plants respond through an intricate network of signal transduction pathways (González-Schain et al., 2016; Wang et al., 2019). In rice, heat stress at the reproductive stage affects pollen viability and spikelet fertility, thereby reducing the grain yield. Pollen viability is reduced primarily due to pollen desiccation and denaturation of proteins. Spikelet sterility is attributed to poor anther dehiscence and reduced pollen production, reducing the number of viable pollens reaching the stigma (Matsui et al., 2000, 2001; Prasad et al., 2006). Additionally, tight closure of anther locules by cell layers could also hinder anther dehiscence (Matsui and Omasa, 2002). This is further exacerbated by impaired stigma receptivity due to its exertion out of the spikelet into hot ambience (Wu and Yang, 2019). However, the female reproductive organ is comparatively more resilient to heat stress compared to the male counterpart. Post-fertilization, grain filling, and maturation are equally sensitive to heat stress. High temperature reduces the grain filling period but hastens the grain maturation rate, leading to the impairment of grain filling and resulting in poorly filled chalky grains. These grains break easily on hulling and milling, affecting the head rice recovery in rice (Lyman et al., 2013).

Breeding rice for heat stress tolerance is one of the viable options for mitigating the ill effects of heat stress in rice. Genetic variability for tolerance to reproductive stage heat stress (RSHS) in rice has been well documented (Tenorio et al., 2013; Bheemanahalli et al., 2016; Pradhan et al., 2016; Cheabu et al., 2019; de Brito et al., 2019; Ravikiran et al., 2020). Various mechanisms conferring reproductive stage heat stress tolerance (RSHT) in rice have been reported, which include 1) escape through early morning flowering (Ishimaru et al., 2010; Julia and Dingkuhn, 2013; Hirabayashi et al., 2014), 2) avoidance through mainly evaporative cooling (Julia and Dingkuhn, 2013), and 3) tolerance (Jagadish et al., 2010). True tolerance is primarily adjudged through spikelet fertility, grain yield, and stress tolerance indices. Both spikelet fertility and grain yield under RSHS are quantitative traits, and a large number of QTLs governing RSHT have been documented (Ravikiran et al., 2020). Two major QTLs, *qHTSF1.1* and *qHTSF4.1*, for spikelet fertility were reported on chromosomes 1 and 4 from an upland *aus* cultivar, Nagina 22 (Ye et al., 2012). *qHTSF4.1* has been fine-mapped to around 1.2 Mb region using BC_5_F_2_ population, and its effect was validated in a set of 24 rice varieties and different genetic backgrounds (Ye et al., 2015a; b). However, in the majority of the studies for mapping RSHT, the phenotypic variance accounted for by the QTLs identified is very low. Furthermore, most of them are neither validated nor finely mapped and cannot be reliably utilized for marker assisted selection for RSHT in rice. Several putative candidate genes associated with heat stress tolerance such as *TOGRI* (*Thermotolerant Growth Required 1*) (Wang et al., 2016), *SLG1* (*Slender guy 1*) (Xu et al., 2020), and *psbA* (Chen et al., 2020) in addition to *OgTTI1* (*Thermo Tolerance 1*) (Li et al., 2015) encoding the *α*_2_ subunit of 26s proteasome, heat shock proteins and heat shock transcription factors, have been proposed in rice.

A biparental mapping population generally limits the number of genes that can be detected as its genetic variation is restricted between the contrasting parents, both phenotypic and genotypic. This would essentially lead to a large number of key genes/alleles contributing to the variability of a particular trait going unaccounted. Furthermore, the number of false positives in linkage mapping is higher due to the existence of extensive genomic regions under disequilibrium. Genome-wide association studies (GWAS) provide a valuable alternative to linkage mapping since it is based on historic recombination, which considerably breaks the linkage disequilibrium (LD) blocks and also enhances the resolution of QTL detected. It is a particularly useful tool to dissect complex traits such as heat stress tolerance. GWAS is popular in rice as it is endowed with vast genetic variability conserved in gene banks and access to rich genomic resources. Recently, pan-genome data of 67 diverse rice accessions with 16.5 million SNPs, 5.5 million indels, and 0.9 million structural variants have been made available (Zhao et al., 2018). Several SNP arrays with varying densities already exist in rice enabling high-throughput genotyping, which include 44 K (Zhao et al., 2011), 6 K (Yu et al., 2014), 50 K (Singh et al., 2015), 700 K (McCouch et al., 2016), and 7 K (Thomson et al., 2017). GWAS is routinely utilized for mapping biotic (Li et al., 2019; Hada et al., 2020), abiotic (Rohilla et al., 2020; Yuan et al., 2020), and grain quality (Misra et al., 2019; Tang et al., 2019)-related attributes in rice. Even though vast genetic variation was reported for RSHT in rice, it has not been properly utilized for identifying MTAs through a GWAS. There is only one systematic GWAS effort which compares three strategies of association mapping (Lafarge et al., 2017). Hence, in the present study, GWAS for RSHT was carried out through the phenotypic characterization of a set of 192 rice accessions for RSHT in terms of spikelet fertility and grain yield and by genotyping through 50K SNP markers, which lead to the identification of significant marker trait associations (MTAs) through three models. The SNPs associated with the significant MTAs are identified as quantitative trait nucleotides (QTNs). Furthermore, the candidate genes governing RSHT in the vicinity of these MTAs were also identified and discussed.

## Materials and Methods

### Phenotypic Characterization of Germplasm for Reproductive Stage Heat Stress

The present study was conducted on a set of 192 diverse rice genotypes assembled from a germplasm collection maintained at the Division of Genetics, ICAR–Indian Agricultural Research Institute (ICAR-IARI). The experiment was conducted at the IARI–Rice Breeding and Genetics Research Center, Aduthurai, Tamil Nadu (11° 00′N; 79° 28′E, 19.5 m) during the late *Rabi* (December–April) season of 2018–19. The crop was raised under irrigated transplanted conditions. The genotypes were laid out in an augmented randomized complete block design with four blocks – 48 genotypes along with five checks were randomly allocated to 53 plots per block. In each plot, genotypes were planted in three rows with a spacing of 20 × 15 cm. Two staggered sowings with a gap of 30 days were completed to adjust the flowering time of the germplasm to the targeted seasonal temperatures. The first sowing was completed in the second fortnight of December, which served as the unstressed control since the peak anthesis of the genotypes coincided with optimum (max) temperatures, 33–35°C. The second staggering was taken up in the second fortnight of January, which served as reproductive heat stress treatment wherein peak anthesis of genotypes occurred at higher ambient temperatures, that is, 39–41°C. There were no differences in other agronomic practices in both staggered experiments, and all necessary care was taken to raise a healthy rice crop.

### Observations Recorded and Data Analysis

The genotypes from two staggered sowings were closely monitored particularly for their flowering and anthesis to make sure that there are no escapes due to variation in flowering duration in these genotypes. At physiological maturity, five randomly selected plants from the middle row of every plot were harvested separately. One panicle from the main tiller of each genotype was sampled for spikelet fertility. The plants were then threshed separately and weighed to record data on a single plant yield. For spikelet fertility, the panicles were threshed individually, and the filled and unfilled grains were counted manually. The proportion of filled grains among the total number of grains per panicle was expressed as spikelet fertility percent. Additionally, the stress tolerance index (STI; Fernandez, 1992) was calculated for both grain yield per plant and spikelet fertility using the following formula:

$$\frac{\left(Ys\right)\left(Yp\right)}{\bar{Y}p^{2}},$$

where Y*s* and Y*p* are the average yield/spikelet fertility of genotypes under stressed and unstressed conditions, respectively, while Y*p* represents the mean yield/spikelet fertility of all genotypes under unstressed conditions. Statistical analysis of the phenotypic data was conducted using R statistical software by utilizing appropriate packages. The adjusted means from augmented RCB analysis were generated using the *agricolae* package run on the R studio (RStudio Team, 2016). The package *ggplot2* was utilized to draw frequency curves for different traits.

### SNP Genotyping and Filtering

The SNP genotyping of the germplasm set carried out in an earlier study (Bollinedi et al., 2020) was utilized for this study as well. Briefly, 2-week-old seedlings from the nursery were sampled and processed in liquid nitrogen. DNA was extracted using the cetyl trimethyl ammonium bromide method (Murray and Thompson, 1980). DNA quality was first assessed on 0.8% agarose gel, which was further confirmed using a nanospectrophotometer (NanoDrop™ 2000/2000c, Thermo Fisher Scientific, DE, United States). DNA samples were then sent for SNP genotyping using 50k Affymetrix GeneChip (Thermo Fisher Scientific, United States). The technical details of this custom-made gene chip were explained in Singh et al. (2015). The array houses 50,051 SNPs selected from 18,980 genes covering 12 rice chromosomes with an interval of 1 kb between two adjacent SNPs. The genotyping data of 50,051 SNPs were first filtered for rare alleles with a minor allele frequency cutoff of 5%, and then for missing values, markers with >20% missing reads were dropped. The final number of markers utilized for downstream analysis was reduced to 32,712 SNPs.

### Population Structure, Linkage Disequilibrium, and Association Analysis

The population structure of the germplasm and LD decay was worked out as explained in Bollinedi et al. (2020). However, principal component analysis (PCA), inbuilt in the R platform for association analysis, genome association, and prediction integrated tool (GAPIT), was conducted to cross-check the number of subpopulations reported (Lipka et al., 2012). The scree plot generated from PCA was utilized to decide the number of components explaining the optimum population structure and thereby the number of subpopulations. Linkage disequilibrium was estimated based on squared allele frequency correlations (*r* ^*2*^) with significant *p* values (<0.05) for each pair of loci. LD decay was depicted using bins of 200 kb, and the average *r* ^*2*^ value was plotted against the physical distance. The distance at which the *r* ^*2*^ value plummeted to half of its average maximum value was considered as the rate of LD decay. The association analysis was conducted in GAPIT by executing three different models—mixed linear model (MLM), fixed and random model circulating probability unification (FarmCPU), and Bayesian-information and linkage-disequilibrium iteratively nested keyway (BLINK). Phenotypic data generated under both normal and stressed conditions were used for association analysis. The significant threshold for marker trait associations (MTAs) was fixed at −log_10_ *p* > 5.8 (Bonferroni threshold) to avoid type 1 errors (false positives). However, to prevent type 2 errors (false negatives), the threshold was relaxed to −log10 *p* > 5.0, wherever appropriate (Melandri et al., 2020). For every significant MTA, quantitative trait nucleotide (QTN) was identified.

### Co-Localized QTLs, Candidate Genes, and Their *In Silico* Expression Analysis

The physical positions of the MTAs in the rice genome were further analyzed for the presence of any reported QTLs for reproductive stage heat stress tolerance, and MTAs which did not co-localize with QTLs mapped in earlier studies were considered novel. The candidate genes in and around these MTAs were identified using the genome browser of the Rice Genome Annotation Project (http://rice.uga.edu/cgi-bin/gbrowse/rice). The tissue-specific expression of the putative candidates identified was analyzed using the datasets available on the RiceXPro website (https://ricexpro.dna.affrc.go.jp). Furthermore, the candidate genes were compared with the results of previous transcriptomic studies and the expression dynamics of common genes.

## Results

### Phenotypic Characterization of the Germplasm for Reproductive Stage Heat Stress Tolerance

**FIGURE 1:**
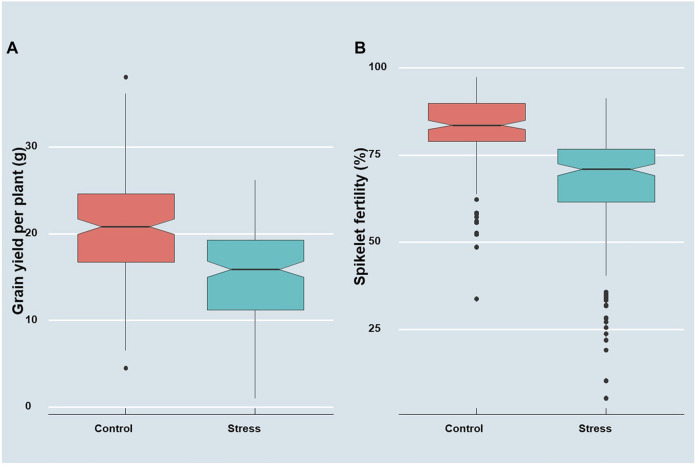
Boxplots depicting the distribution of **(A)** grain yield plant^−1^ (g) and **(B)** spikelet fertility (%) under control and heat stress conditions.

A preliminary augmented ANOVA revealed significant test genotype effects, check effects, and checks versus test entry effects for both grain yield and spikelet fertility, particularly under heat stress (Supplementary Table S1). A significant difference was observed in the diurnal mean temperatures during the peak anthesis stage of the germplasm between the two staggered sowing windows. The temperature range during anthesis for the first staggered sown set was between 33 and 35°C, whereas it was 38–40°C for the late sown set (Supplementary Figure S1 and Figure 1 of Ravikiran et al., 2020). As a result, the performance of the genotype showed a significant reduction in the late sown set (Figure 1). A mean reduction of 26% was observed for grain yield, while it was 19% for spikelet fertility in the late sown set exposed to high-temperature stress at the reproductive stage. The grain yield and spikelet fertility ranged from 4.47 to 38.13 g and from 33.85 to 97.52%, respectively, under the timely sown unstressed situation. However, the grain yield under heat stress ranged from 1.04 to 26.23 g, while the spikelet fertility ranged from 5.30 to as high as 91.46% (Table 1). According to IRRI Standard Evaluation System (SES; IRRI, 2002), 27 genotypes are highly sterile with SF of <50%, 96 genotypes were partially sterile with SF ranging between 50 and 74%, 65 genotypes were fertile with SF varying between 75 and 89%, and the remaining four genotypes were highly fertile with spikelet fertility of ≥90% under heat stress conditions. For grain yield under heat stress, the genotypes Bhubana (26.24 g), Indravati (25.56 g), PRR127 (25.42 g), and PRR122 (24.93 g) were found to be superior, while for spikelet fertility, DV85 (91.46%), BJ1 (90.18%), and NDR359 (90.35%) were found to be the best. Grain yield under heat stress and spikelet fertility under the unstressed control showed high broad sense heritability. Both grain yield and spikelet fertility under heat stress followed near normal distributions (Shapiro–Wilk’s *p*-value > 0.05). Furthermore, the range of stress tolerance index (STI) calculated for grain yield (STIGY) (0.01–2.14 with a mean of 0.81) was higher than that of spikelet fertility (STISF) (0.03–1.27 with a mean of 0.82).

**TABLE 1:** Summary statistics of grain yield plant^−1^ and spikelet fertility under control and reproductive stage heat stress conditions.

Statistics	Grain Yield plant^−1^ (g)		Spikelet Fertility (%)		STI_sf_	STI_gy_
	Control	Heat Stress	Control	Heat Stress		
Mean	20.41	15.14	82.49	66.82	0.82	0.81
Min	4.47	1.04	33.85	5.30	0.03	0.01
Max	38.13	26.23	97.52	91.46	1.27	2.14
S.D.	6.60	5.77	9.87	16.85	0.25	0.47
S.E.	0.47	0.42	0.72	1.22	0.01	0.03
C.V. (%)	24.05	16.28	6.30	23.09	30.54	58.25
PCV	31.93	37.62	11.72	24.75		
GCV	20.89	33.94	9.89	16.54		
h^2^ (broad sense)	42.83	81.36	71.19	65.91		
GA	5.78	9.58	14.21	10.08		

S.D., standard deviation; S.E., standard error of the mean; C.V., coefficient of variation; PCV, phenotypic coefficient of variation; GCV, genotypic coefficient of variation; h^2^, heritability; GA, genetic advance; STI_sf_, stress tolerance index calculated for spikelet fertility; STI_gy_, stress tolerance index calculated for grain yield per plant.

### Population Structure and Linkage Disequilibrium

**FIGURE 2:**
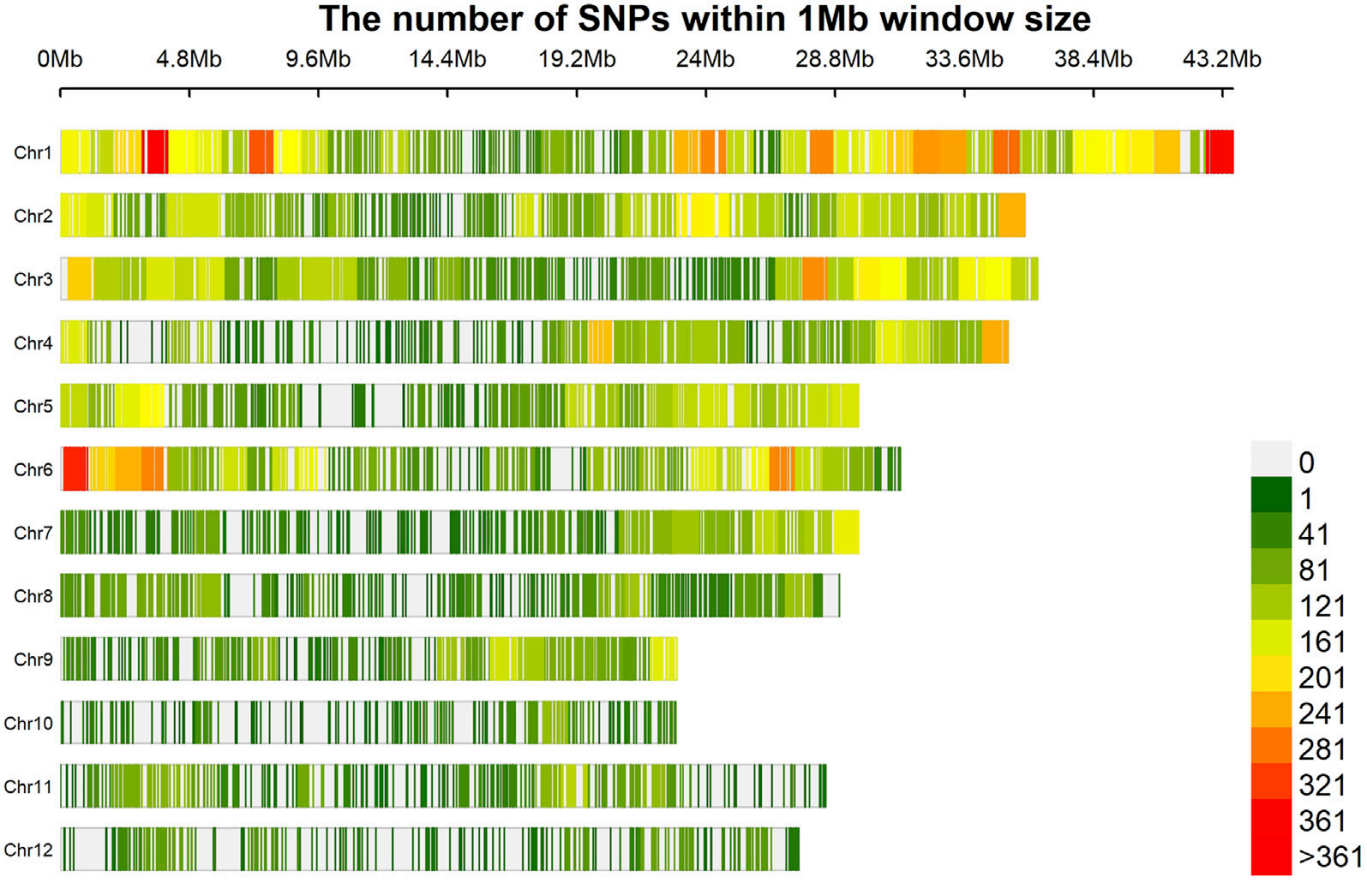
Density of SNP across the genome. The highest coverage can be observed on chromosomes 1 and 6, while the lowest is on chromosome 12.

**FIGURE 3:**
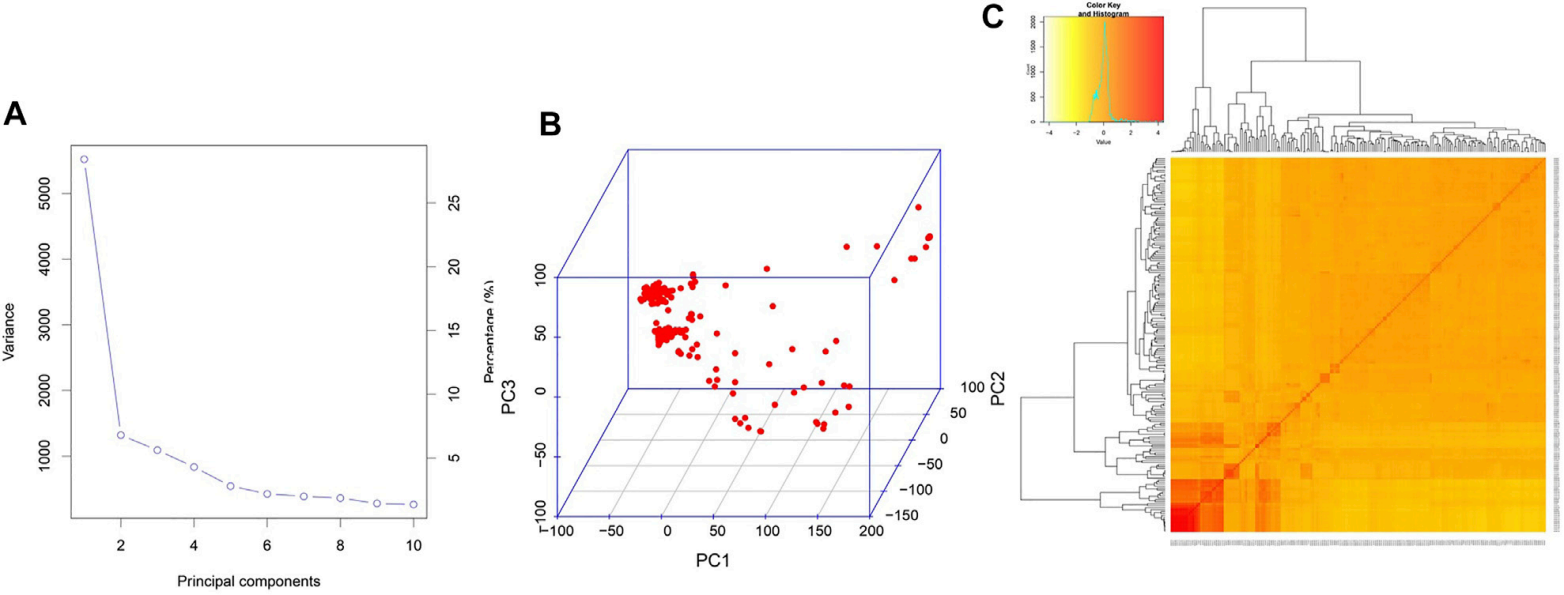
**(A)** Scree plot depicting the proportion (%) of total variance captured by various principal components. **(B)** PCA 3D plot illustrating the distribution of genotypes across three principal components. **(C)** Pair-wise kinship heat map between the genotypes. The figure in the inset describes the color code of the heat map and the frequency curve of kinship values among the genotypes.

The marker density plot showed the coverage of markers with an average distance of around 30 kb with almost 90% of the markers spaced within a 5 kb distance (Figure 2). In addition, the majority of the genotypes (>150) and markers (>25,000) showed the least heterozygosity, reflecting the true breeding nature of genotypes. The highest *r* ^*2*^ (>0.8) was obtained with a genomic span of <5 kb, followed by a sudden dip (50%) at around 200 kb. Although there were many peaks and valleys, beyond this, there was a gradual general decline in LD which reached less than 0.1 at around 400 kb. Considering this, the marker coverage obtained in the present study is adequate and hence can be utilized for association analysis. Two covariates, population structure and kinship, were employed to cull out false positives. As described earlier (Bollinedi et al., 2020), the population structure analysis using STRUCTURE based on the graph drawn between ∆K and K values showed the existence of three subpopulations, denoted as POP1, POP2, and POP3. This was further predicted by principal component analysis conducted in the present study. The scree plot showed that a significant portion of variance was captured by PC1 itself (29%), followed by PC2 (7%) and PC3 (6%) (Figure 3A). Beyond PC3, the variation contributed by individual PCs was meager (<5%) and can be safely ignored. Hence, a PCA 3D plot between PC1, PC2, and PC3 was considered for interpreting the subpopulation composition of the association panel (Figure 3B). One of the subpopulations possessed the maximum number of genotypes (139 genotypes), followed by the second subpopulation with 35 accessions and the third with 15 genotypes. The first subpopulation with the maximum membership is composed of most of the popular rice varieties of the country such as MTU1001, ADT 39, MAS946-1, Improved Sabarmati, and some advanced breeding lines. The second subpopulation is made of unique temperate rice landraces of Jammu and Kashmir. Furthermore, the heatmap of a kinship value revealed that the maximum number of kinship values populated around 0 to 0.5, indicating a very weak relatedness or maximum genetic diversity in the association panel utilized in the present study (Figure 3C).

### Genome-Wide Association Analysis

**FIGURE 4:**
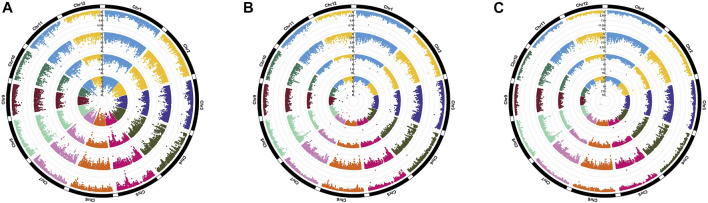
Circular Manhattan plots depicting significant MTAs using MLM **(A)**, FarmCPU **(B)**, and BLINK **(C)** for various traits. From outside to inside: grain yield per plant (GYPP) under stress, spikelet fertility (SF) under stress, STI calculated for GYPP, and STI calculated for SF.

**FIGURE 5:**
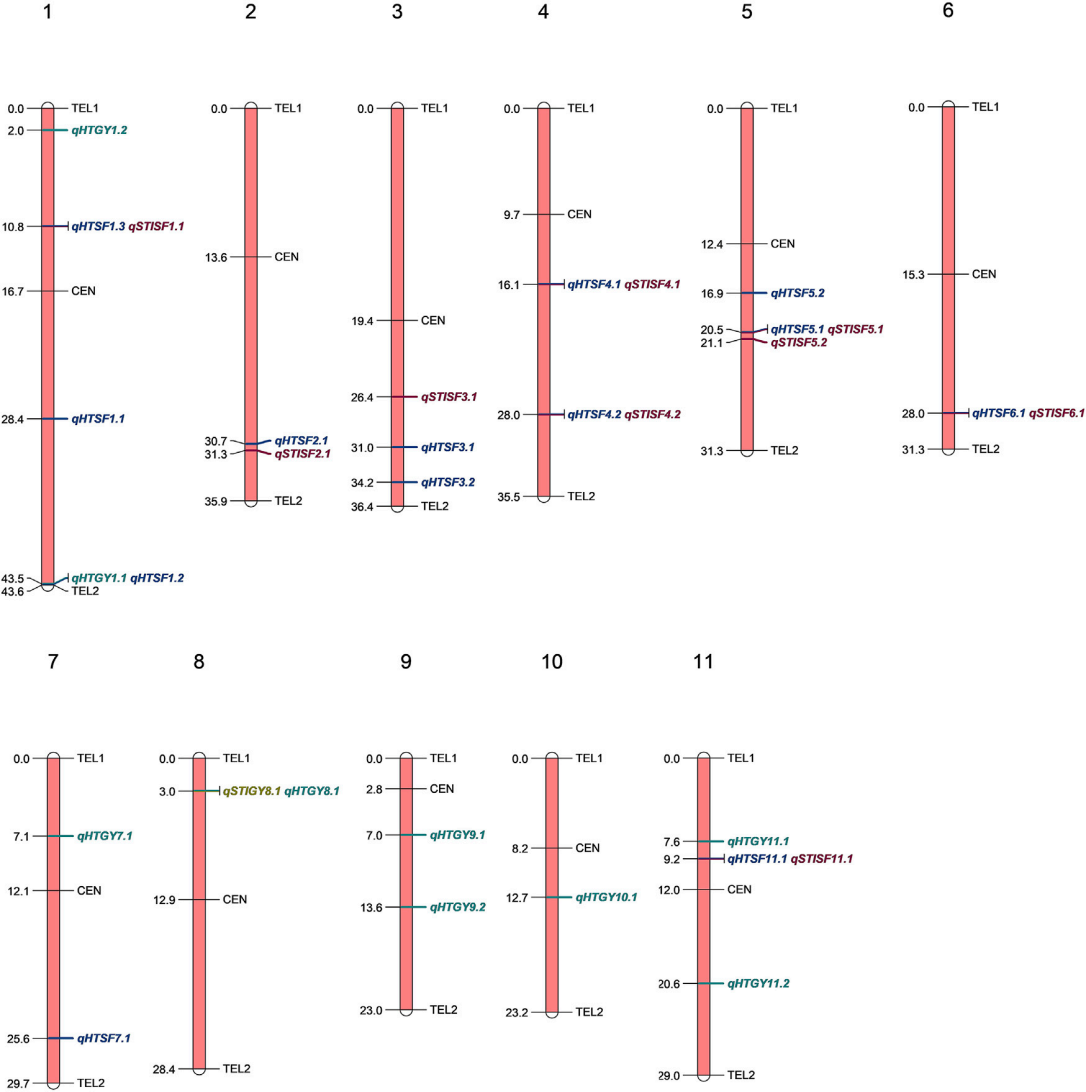
Physical positions (Mb) of various MTAs identified across the rice genome through various models employed in GAPIT.

**FIGURE 6:**
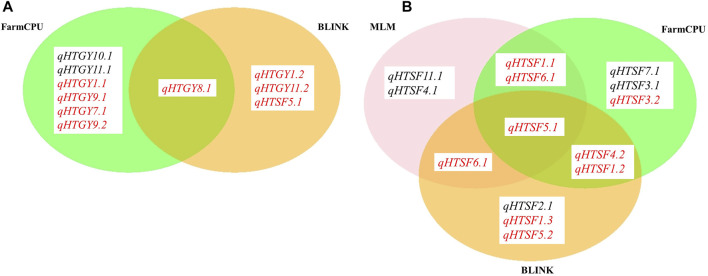
MTAs identified through MLM, FarmCPU, and BLINK models for **(A)** GYPP and **(B)** SF. Novel MTAs are highlighted in red.

Genome-wide association analysis was performed by executing three different models, namely, MLM, FarmCPU, and BLINK. The significant MTAs obtained from heat-stressed conditions are summarized in Table 2 along with the corresponding Manhattan plots (Figure 4). However, no significant MTAs could be detected under normal unstressed conditions. The locations of the QTNs identified are depicted in Figure 5, and common QTNs across the models are shown in Figure 6. For grain yield, MLM failed to detect any significant association, while seven QTNs were detected through FarmCPU and three through BLINK. Only one MTA, *qHTGY8*.*1*, was common between these two models (Figure 6A). This MTA registered the highest probability through BLINK, while it was relatively lower through FarmCPU, but high *R* ^2^ values through both models. The MTA *qHTGY10.1* showed the highest probability under FarmCPU. MTAs with higher probability values, *qHTGY10.1* and *qHTGY11.1*, were also reported earlier but for other traits. For spikelet fertility, the highest number of MTAs was detected using the FarmCPU model (8), followed by MLM (7) and BLINK (7). Among these, one MTA was identified consistently across three models, *qHTSF5.*1 (Figure 6B), which also displayed the highest *R* ^2^ value (0.10). Furthermore, *R* ^2^ values of MTAs identified through MLM are slightly higher than those identified through the other two models. This reflects that MLM lays more emphasis on major QTLs and may miss some minor QTLs which play an equally important role in trait expression. The majority of these MTAs are novel in terms of RSHT except for five MTAs, which coincided with the positions of previously reported MTAs. The MTAs identified for STI are almost the same as that of original trait values, reflecting a high correlation between the two. Particularly under MLM, a group of MTAs clustered in the region 20.5 Mb. This region showed a significant hit with FarmCPU and BLINK as well.

**TABLE 2:** Significant MTAs identified for grain yield plant^−1^ (GYPP) and spikelet fertility (SF) under heat stress and their respective stress tolerance indices.

Trait	SNP	Model	Fav Allele	Chr	Position	Probability	*R* ^2^	MTA	Previously Reported QTLs^a^
GYPP	AX-95938448	FarmCPU	C	10	12,691,143	9.11E-08	0.02	*qHTGY10.1*	*qhr3-1* Cao et al. (2003)
	AX-95938539		T	11	7,598,615	2.03E-07	0.05	*qHTGY11.1*	*qADL09-11* Tazib et al. (2015)
	AX-95918021		G	1	43,529,356	2.74E-07	0.03	*qHTGY1.1*	-
	AX-95959896		C	9	6,976,331	4.10E-06	0.02	*qHTGY9.1*	-
	AX-95957982		G	7	7,144,612	2.51E-05	0.02	*qHTGY7.1*	-
	AX-95938211		A	9	13,559,420	4.95E-05	0.02	*qHTGY9.2*	-
	**AX-95937704**		**C**	**8**	**3,014,640**	**8.57E-05**	**0.05**	***qHTGY8.1***	-
	**AX-95937704**	BLINK	**C**	**8**	**3,014,640**	**2.43E-10**	**0.05**	***qHTGY8.1***	-
	AX-95916113		C	1	2,051,081	8.53E-07	0.04	*qHTGY1.2*	-
	AX-95932,408		C	11	20,582,446	2.31E-05	0.03	*qHTGY11.2*	-
SF	AX-95926541	MLM	C	5	20,542,291	1.62E-06	0.10	*qHTSF5.1*	-
	AX-95961044		G	11	9,168,125	1.09E-05	0.08	*qHTSF11.1*	*qHTSF11*.2 Ye et al. (2015a) *qLD10*-11 Tazib et al. (2015)
	**AX-95926170**		**G**	**5**	**20,547,603**	**1.70E-05**	**0.08**	***qHTSF5.1***	-
	**AX-95927077**		**T**	**5**	**20,540,979**	**1.70E-05**	**0.08**	***qHTSF5.1***	-
	AX-95962,696		C	4	16,049,320	1.73E-05	0.08	*qHTSF4.1*	*SSPF4* Xiao et al. (2011a) *qPF4* Xiao et al. (2011b)
	AX-95918542		A	1	28,440,408	4.95E-05	0.07	*qHTSF1.1*	-
	AX-95956527		T	6	28,019,302	5.42E-05	0.07	*qHTSF6.1*	-
	AX-95956527	FarmCPU	T	6	28,019,302	2.53E-10	0.07	*qHTSF6.1*	-
	**AX-95926541**		**C**	**5**	**20,542,291**	**3.57E-09**	**0.10**	***qHTSF5.1***	-
	AX-95930097		G	7	25,603,077	6.96E-08	0.03	*qHTSF7.1*	*qAL10*-7 Tazib et al. (2015)
	AX-95924814		C	4	28,011,513	9.78E-08	0.04	*qHTSF4.2*	-
	AX-95944879		T	1	28,469,004	6.75E-06	0.06	*qHTSF1.1*	-
	AX-95923670		G	3	31,000,572	1.45E-05	0.03	*qHTSF3.1*	*qtl_3.4* Jagadish et al. (2010)
	AX-95918181		G	1	43,540,685	2.80E-05	0.04	*qHTSF1.2*	-
	AX-95949687		A	3	34,204,030	5.32E-05	0.04	*qHTSF3.2*	-
	**AX-95926541**	BLINK	**C**	**5**	**20,542,291**	**2.15E-10**	**0.10**	***qHTSF5.1***	-
	AX-95924814		C	4	28,011,513	7.73E-10	0.04	*qHTSF4.2*	-
	AX-95921100		T	2	30,694,562	2.67E-09	0.03	*qHTSF2.1*	*qtl_2.2* Jagadish et al. (2010)
	AX-95940947		T	1	10,799,807	5.56E-09	0.05	*qHTSF1.3*	-
	AX-95918181		G	1	43,540,685	3.66E-07	0.04	*qHTSF1.2*	-
	AX-95936404		C	5	16,852,857	4.32E-05	0.05	*qHTSF5.2*	-
	AX-95956527		T	6	28,019,302	6.21E-05	0.07	*qHTSF6.1*	-
STIGY	AX-95930775	FarmCPU	A	8	3,009,287	8.96E-05	0.03	*qSTIGY8.1*	-
	AX-95930775	BLINK	A	8	3,009,287	8.96E-05	0.03	*qSTIGY8.1*	-
STISF	AX-95926541	MLM	C	5	20,542,291	5.10E-07	0.11	*qSTISF5.1*	-
	**AX-95927077**		**T**	**5**	**20,540,979**	**5.11E-06**	**0.09**	***qSTISF5.1***	-
	**AX-95926170**		**G**	**5**	**20,547,603**	**5.11E-06**	**0.09**	***qSTISF5.1***	-
	AX-95952,837		A	5	20,642,828	8.34E-06	0.09	*qSTISF5.1*	-
	AX-95961044		G	11	9,168,125	1.53E-05	0.08	*qSTISF11.1*	*qHTSF11.2* Ye et al. (2015a) *qLD10-11* Tazib et al. (2015)
	AX-95927241		A	5	21,126,325	4.49E-05	0.07	*qSTISF5.2*	-
	AX-95962,696		C	4	16,049,320	4.54E-05	0.07	*qSTISF4.1*	*SSPF4* Xiao et al. (2011a) *qPF4* Xiao et al. (2011b)
	**AX-95926541**	FarmCPU	**C**	**5**	**20,542,291**	**3.77E-09**	**0.11**	***qSTISF5.1***	-
	AX-95924814		C	4	28,011,513	1.48E-07	0.03	*qSTISF4.2*	-
	AX-95940947		G	1	10,799,807	1.17E-06	0.04	*qSTISF1.1*	-
	AX-95919771		T	2	31,278,025	6.76E-06	0.03	*qSTISF2.1*	-
	**AX-95926541**	BLINK	**C**	**5**	**20,542,291**	**8.87E-15**	**0.11**	***qSTISF5.1***	-
	AX-95924814		C	4	28,011,513	1.50E-05	0.03	*qSTISF4.2*	-
	AX-95956527		T	6	28,019,302	3.67E-05	0.06	*qSTISF6.1*	-
	AX-95919771		T	2	31,278,025	5.25E-05	0.03	*qSTISF2.1*	-

a QTLs, reported exclusively for reproductive stage heat stress tolerance in rice; Chr, chromosome; GYPP, grain yield per plant; SF, spikelet fertility; STISF, stress tolerance index calculated for spikelet fertility; STIGY, stress tolerance index calculated for grain yield per plant; MTAs which are common across two to three models are highlighted in bold for various traits.

### Allelic Effects of Major Quantitative Trait Nucleotides Identified

**FIGURE 7:**
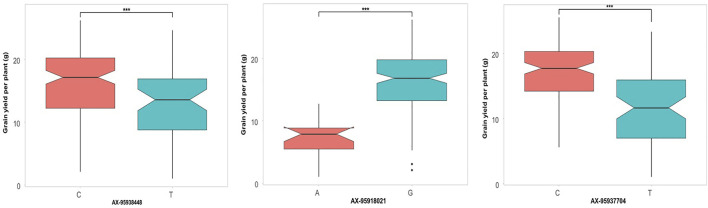
Comparison of allelic effects of major QTNs identified in the present study for grain yield per plant under reproductive stage heat stress.

**FIGURE 8:**
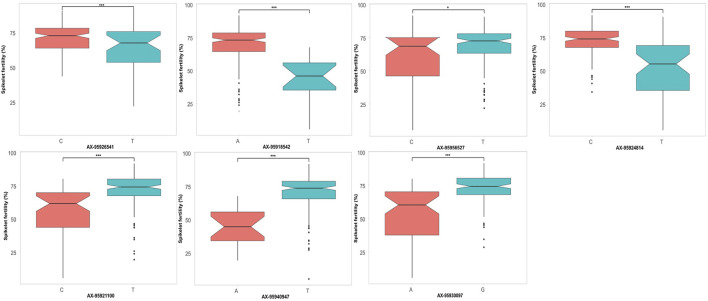
Comparison of allelic effects of major QTNs identified in the present study for spikelet fertility under reproductive stage heat stress.

Additionally, the allelic effects of a subset of QTNs identified through GWAS showing significant effects on the respective trait were inspected. For grain yield (Figure 7), three QTNs were selected, namely, *qHTGY10.1*, *qHTGY1.1*, and *qHTGY8.1*, with the linked SNP AX-95938448, AX-95918021, and AX-95937704, respectively. Among these three markers, the greatest significant difference for the grain yield per plant between the genotypes carrying alternate alleles was found for the marker AX-95918021, followed by AX-95937704 and AX-95938448. The genotypes carrying the ‘A’ allele for AX-95918021 showed a mean grain yield of 8 g, while those carrying its alternate allele, ‘G’, had a mean grain yield of 18 g. Similarly, genotypes with the ‘C’ allele of AX-95937704 exhibited a grain yield of 12 g, while those carrying the ‘T’ allele showed a grain yield of 18 g. Similarly, for spikelet fertility, seven major QTNs were selected, among which AX-95940947 with its linked QTN *qHTSF1.3* showed a highly significant difference in spikelet fertility values for the two alternate alleles closely followed by AX-95918542 linked to QTN *qHTSF1.1* (Figure 8). The ‘A’ allele of AX-95940947 showed an allelic effect in terms of spikelet fertility (%) of 47, while the ‘T’ allele showed an effect of 75. The ‘A’ allele of AX-95918542 exhibited an effect of 75% spikelet fertility, and the ‘T’ allele showed an effect of 50%. Interestingly, both these QTNs are located on chromosome 1 but 18 Mb apart. The remaining QTNs also showed statically significant (*p* < 0.001) differences between the trait values conferred by their respective alleles, except AX-95956527, with its linked QTN, *qHTSF6.1*, significant at only *p* < 0.05. This further attests to the robustness of major QTNs identified in the present study.

### Identification of Putative Candidate Genes for RSHT and Their In Silico Expression Analysis

A total of 11 candidate genes were identified near 10 SNPs associated with grain yield under heat stress distributed on chromosomes 1 (2), 7 (1), 8 (3), 9 (2), 10 (1), and 11 (2) (Table 3). Of these genes, five of them are kinases–diacylglycerol kinase, Ser/Thr protein kinase, and receptor-like kinase involved in stress signaling cascades. Others include glucosylceramidase, zinc ion binding protein, DUF1336 domain-containing protein, cytochrome P450, and lipase with putative roles in plant defense responses. Similarly, 17 candidate genes were found in the vicinity of 10 SNPs associated with spikelet fertility under heat stress scattered across chromosomes 1 (2), 2 (3), 3 (5), 4 (1), 5 (3), 6 (1), 7 (1), and 11 (1). The majority of these genes are involved in protein processing and protein–protein interactions such as tetratricopeptide repeat domain-containing protein, an E3 ubiquitin ligase, BTB/POZ domain-containing protein, U-box domain-containing protein, ankyrin repeat-containing protein, and RING zinc finger protein. Others include genes involved in abiotic stress responses and other essential pathways of plant development. The survey of expression database (RiceXPro) revealed that the majority of the putative candidate genes identified for grain yield showed predominant vegetative stage-specific expression, whereas those identified for spikelet fertility expressed primarily in the reproductive organs (Supplementary Figures S3A,B). Additionally, the putative candidate genes identified in the present study were compared with the differentially expressed genes (DEGs) identified in previous transcriptomic studies under heat stress in rice. Six genes were found common with the DEGs reported by Liu et al. (2020) in the thermo-tolerant genotype, SDWG005 (Table 4). Of these, three genes were upregulated, while three genes were downregulated under heat stress. There were four putative candidate genes that matched with the DEGs observed by Cai et al. (2020), of which two genes were upregulated and the other two were downregulated.

**TABLE 3:** Candidate genes in the QTN regions identified through three GWAS models for grain yield per plant, spikelet fertility, and their respective stress tolerance index.

Trait or gene ID	Closest SNP	Chr	Start	End	Gene Annotation	Putative Gene Function
**Grain yield plant** ^−1^						
LOC_Os10g24690	AX-95938448	10	12,690,416	12,689,328	Expressed protein	-
LOC_Os11g13810	AX-95938539	11	7,595,274	7,606,870	Non-lysosomal glucosylceramidase, putative, expressed	Catabolism of sphingolipids
LOC_Os09g12250	AX-95959896	9	6,975,494	6,978,083	Expressed protein	-
LOC_Os07g12520	AX-95957982	7	7,145,539	7,149,827	Zinc ion binding protein, putative, expressed	Reproductive development
LOC_Os09g22450	AX-95938211	9	13,559,548	13,563,071	Lipase, putative, expressed	Disease resistance
LOC_Os08g05640	AX-95937704	8	3,013,718	3,016,330	DUF1336 domain-containing protein	Disease resistance
LOC_Os08g05620	AX-95937704	8	3,007,241	3,009,195	Cytochrome P450, putative, expressed	Regulation of non-enzymatic antioxidant synthesis
LOC_Os08g05650	AX-95937704	8	3,017,356	3,022,047	Diacylglycerol kinase, putative, expressed	Plant stress response
LOC_Os01g04570	AX-95916113	1	2,048,717	2,052,521	Ser/Thr protein kinase, putative, expressed	Heat shock response
LOC_Os01g04580		1	2,053,583	2,057,638	Ser/Thr protein kinase, putative, expressed	
LOC_Os11g35120	AX-95932,408	11	20,589,041	20,591,080	OsWAK116 - OsWAK receptor-like cytoplasmic kinase OsWAK-RLCK, expressed	Signal transduction
**Spikelet fertility**						
LOC_Os11g16540	AX-95961044	11	9,156,589	9,173,338	Tetratricopeptide repeat domain-containing protein, expressed	Protein–protein interactions
LOC_Os01g49470	AX-95918700	1	28,447,887	28,458,977	E3 ubiquitin ligase, putative, expressed	Proteolysis
LOC_Os06g46240	AX-95956527	6	28,007,285	28,017,490	BTB/POZ domain-containing protein, putative, expressed	Transcriptional regulation and protein degradation
LOC_Os07g42750	AX-95930097	7	25,601,642	25,606,513	DDT domain-containing protein, putative, expressed	Chromatin remodeling
LOC_Os04g47170	AX-95924814	4	28,013,719	28,017,003	ATROPGEF7/ROPGEF7, putative, expressed	Secondary cell wall formation
LOC_Os01g49490	AX-95944879	1	28,467,690	28,471,172	Expressed protein	-
LOC_Os03g54084	AX-95923670	3	31,004,724	31,009,782	Phytochrome C, putative, expressed	Photoperiodic response
LOC_Os03g54091		3	31,009,978	31,013,940	OsTOP6A1-Topoisomerase 6 subunit A homolog 1, expressed	Meiotic recombination
LOC_Os03g60140	AX-95949687	3	34,198,836	34,201,827	U-box domain-containing protein, putative, expressed	Protein degradation
LOC_Os03g60130		3	34,194,882	34,197,992	Transcription elongation factor protein, putative, expressed	Regulation of flower induction
LOC_Os03g60150		3	34,202,415	34,206,308	Protein kinase domain-containing protein, expressed	Kinase activity
LOC_Os02g50270	AX-95921100	2	30,695,324	30,698,401	Ankyrin repeat-containing protein, putative, expressed	Protein–protein interactions and protein chaperoning
LOC_Os02g50280		2	30,698,990	30,701,615	Pentatricopeptide, putative, expressed	Abiotic stress responses
LOC_Os02g50290		2	30,701,962	30,703,636	RING zinc finger protein, putative, expressed	Protein ubiquitination
LOC_Os05g28730	AX-95936404	5	16,850,590	16,853,524	Zinc finger, C3HC4 type domain-containing protein, expressed	Signal transduction
LOC_Os05g28720		5	16,845,905	16,848,027	PPR repeat-containing protein, expressed	Abiotic stress responses
LOC_Os05g28740		5	16,859,683	16,860,977	Universal stress protein domain-containing protein, putative, expressed	Several abiotic stresses
LOC_Os03g46640	AX-95949747	3	26,396,243	26,397,459	Deoxyuridine 5-triphosphate nucleotidohydrolase, putative, expressed	Floral organ development
LOC_Os03g46650		3	26,398,523	26,406,544	WD domain, G-beta repeat domain-containing protein, expressed	Signal transduction

Chr, chromosome.

**TABLE 4:** Differential expression of some putative candidate genes identified in the present study from published datasets.

Gene ID	Log_2_FC^a^ (6 h)	Log_2_FC^a^ (12 h)	Log_2_FC^b^ (36°C)	Log_2_FC^b^ (38°C)	Log_2_FC^c^ (36°C)	Log_2_FC^c^ (38°C)
LOC_Os08g05620	−1.51	−1.40	-	-	-	-
LOC_Os01g04580	-4.87	−2.35	-	-	-	-
LOC_Os05g28740	-	−2.12	-	-	-	-
LOC_Os07g42750	2.45	1.65	-	-	-	-
LOC_Os01g04570	1.68	1.48	-	-	-	-
LOC_Os03g54091	1.43	1.12	-	-	-	-
LOC_Os10g24690	-	-	-	-	−1.89	−3.02
LOC_Os05g28730	-	-	1.67	2.18	2.08	2.45
LOC_Os08g05620	-	-	1.66	2.20	−0.17	0.28
LOC_Os03g46640	-	-	−1.36	−2.10	−0.69	−1.02

a Logarithm of fold change values in thermo-tolerant genotype SDWG005 exposed to 6 and 12 h of heat stress during anthesis (Liu et al., 2020).

b Logarithm of fold change values in thermo-tolerant genotype SDWG005 exposed to heat stress (36 and 38°C) during the meiosis stage (Cai et al., 2020).

c Logarithm of fold change values in thermo-sensitive genotype MH101 exposed to heat stress (36 and 38°C) during the meiosis stage (Cai et al., 2020).

### Selection of RSHT Genotypes With Superior Allelic Combination

The promising genotypes with the *per se* trait values of grain yield and spikelet fertility under stress and STI were shortlisted, and the number of positive alleles of 29 putative MTAs in these tolerant genotypes was investigated. A total of 18 genotypes were identified (Table 5). The genotype RIL 10 accumulated positive alleles for all the 29 SNPs coupled with superior grain yield and spikelet fertility under stress. This was followed closely by Selected Sabarmati, Sitwa Dhan, Bhadrakali, Samba Mashuri, Hema, and Haldamuri, which showed positive alleles for 28 SNPs. Similarly, UPRI 2003–45, B6144-MR-6-0-0, and Pant dhan 18 had positive alleles for 27 SNPs, while RNRM 7, Gouri, Indravati, and ADT 39 had positive alleles for 26 SNPs. The genotypes IR 77384–12-35-3-6-7-2-B and JR 75 showed positive alleles for 25 SNPs, while PRR117 showed positive alleles for 24 SNPs.

**TABLE 5:** List of best-performing genotypes in terms of grain yield, spikelet fertility, and their respective tolerance indices and number of positive alleles of 29 MTAs identified for RSHT.

S. No	Genotype	GY_C	GY_H	STI_GY	SF_C	SF_HT	STI_SF	NPA
1	RIL 10	29.87	23.5	1.68	90.59	86.23	1.14	29
2	Selected Sabarmati	21.98	20.38	1.07	86.8	86.79	1.1	28
3	Sitwa Dhan	26.15	24.66	1.54	77.49	75.02	0.85	28
4	Bhadrakali	25.5	21.54	1.31	90.73	89.81	1.19	28
5	Samba Mahsuri	24.23	22.32	1.29	88.64	78.24	1.02	28
6	Hema	24.88	22.22	1.32	86.79	86.01	1.09	28
7	Haldimuri	25.14	23.38	1.4	97.52	88.35	1.26	28
8	UPRI 2003–45	23.32	21.5	1.2	90.86	83.32	1.11	27
9	B6144-MR-6-0-0	16.81	15.33	0.62	83.59	82.6	1.01	27
10	Pant dhan 18	19.98	19.27	0.92	91.15	88.72	1.19	27
11	RNRM 7	24.62	20.25	1.19	90.63	84.11	1.12	26
12	Gouri	27.44	24.9	1.63	78.06	76.81	0.88	26
13	Indravati	27.88	25.56	1.7	83.64	82.81	1.02	26
14	ADT 39	29.21	23.57	1.64	89.85	79.38	1.05	26
15	IR 77384–12-35-3-6-7-2-B	17.23	15.2	0.81	87.58	83.88	1.08	25
16	JR 75	23.58	19.88	1.12	93.18	87.06	1.19	25
17	PRR 117	16.74	14.53	0.58	87.13	85.58	1.09	24
18	IR 70	25.04	22.48	1.34	81.31	80.29	0.96	24

GY_C, grain yield under control; GY_H, grain yield under heat stress; STI_GY, stress tolerance index for grain yield, SF_C, spikelet fertility under control; SF_H, spikelet fertility under heat stress; STI_SF, stress tolerance index for spikelet fertility; NPA, number of positive alleles.

## Discussion

Crop yields and productivity are predicted to suffer acutely in the coming years due to climate change and global warming. Breeding heat-tolerant crop varieties is essential to address crop losses due to heat stress in rice. The present study aimed to map MTAs governing reproductive stage heat stress tolerance in rice using a diverse set of 192 rice germplasm lines. The two sowing windows utilized could adequately distinguish sensitive and tolerant genotypes, providing a unique situation in which two staggered sowings differed only for the heat stress at the reproductive stage. The screening was carried out during the late *rabi* season in Tamil Nadu, where winter is normally felt inconspicuous from normal weather conditions. The heat stress normally occurs at Aduthurai during the end of the rabi season, marking the beginning of summer. The weather change occurs within a span of 30 days, which is a significant shift from the normal temperature to a high temperature. We had two overlapping conditions: 1) one under a normal sowing which provided the most ideal normal weather conditions all throughout the crop period including the reproductive stage and 2) a late sowing that provided a heat stress only at the terminal stage. The highest temperature reached during the flowering of the second sowing was ∼42°C. The overlap of both the sowings was almost 75%, meaning that both the normal and stressed conditions experienced the same environment most of the time, except for a 30-day window. In the first sowing, the window was during the initial stage, whereas in the second sowing, the window coincided with the reproductive stage. Therefore, both the sowings shared a common environmental influence most of the time. Moreover, weather-wise, the initial 30-day window for the first sowing was not different from that of the second sowing. This provides a unique situation, in which the environmental difference is maximized only during the reproductive stage between both the sowings, and hence, the data generated during this stage could be reliable for studying the RSHT among the genotypes. If found unique and reliable, single season data can be utilized for the GWAS study in rice (Melandri et al., 2020). Field-based phenotyping for RSHT was adopted in other studies (Bheemanahalli et al., 2016; Huang et al., 2016; Pradhan et al., 2016; Sukkeo et al., 2017; Cheabu et al., 2019). A similar sowing date (January last week) to expose the genotypes to heat stress was also chosen by Chidambaranathan et al. (2021) and Pradhan et al. (2016). Wide variation was observed for RSHT in terms of both grain yield and spikelet fertility. A similar variability at this scale (>150 genotypes) for RSHT was also reported in previous studies (Tenorio et al., 2013; Bheemanahalli et al., 2016; Pradhan et al., 2016; Cheabu et al., 2019; de Brito et al., 2019). From a set of 182 *indica*, *japonica*, and *indica*/*japonica* genotypes assessed for RSHT, two genotypes, LTB 14301 and BRS Pampa, were found tolerant (de Brito et al., 2019). Out of 198 genotypes exposed to heat stress, 15 genotypes showed spikelet sterility of <15% (Chidambaranathan et al., 2021). Similarly, from 169 rice accessions evaluated under very high temperatures (40–45°C), four genotypes, namely, AUS17, M9962, SONALEE, and AUS16, were found to be tolerant (with a seed-setting rate of >75%) along with N22. In another study, a total of 240 genotypes were assessed for RSHT at Cuttack, of which 59 genotypes including N22 showed spikelet fertility >60% (Pradhan et al., 2016). From a total of 511 rice genotypes evaluated in an open field, 200 genotypes showing high spikelet fertility (>60%) were further assessed in a controlled environment (38°C), of which 28 genotypes with high fertility were finally selected as donors of RSHT (Tenorio et al., 2013). There were certain genotypes, such as Bhuban, PRR127, DV85, and Improved Samba Mashuri, which could maintain exceptionally higher yields and spikelet fertility under high temperature and can be valuable donors for RSHT after further validation. Grain yield suffered more than spikelet fertility, implying the role of other traits in addition to spikelet fertility which contributes to grain yield under heat stress. Both spikelet fertility and grain yield showed quantitative inheritance qualifying for a GWAS. Considering both phenotypes and genotypes (positive alleles of significant MTAs), RIL 10 was found to be the best performer of all the genotypes and can be a potential heat-tolerant donor after validation in further studies.

In the present study, a total of 32,712 SNPs were utilized for GWAS, which is greater than previous reports. PCA-based population structure analysis showed the presence of three subpopulations as in the previous report (Bollinedi et al., 2020). Assessing the population structure is indispensable, particularly in rice, since it is a self-pollinated crop with clearly differentiated ecotypes—*indica, temperate japonica, tropical japonica, aus/boro,* and *Basmati/Sadri* (Chen et al., 2019). The classification of genotypes through structure analysis in the present study is mainly based on the degree of their domestication and improvement through breeding to which they had been exposed with popular varieties subsumed in one group, with the landraces in the other. Another important parameter to be considered for GWAS is familial relatedness. The kinship values between different genotype pairs clustered around near-zero values, reflecting optimum genetic diversity between the genotypes. Several models are available to conduct the GWAS study. Broadly, they are classified into two categories—single locus models and multi-locus models. Single locus methods test MTAs one SNP at a time akin to single-marker analysis or simple interval mapping of the QTL study. These include the general linear model (GLM) (fits only population structure as a covariate in the model), mixed linear model (MLM) (Zhang Y-M. et al., 2005; Yu et al., 2006) (uses both ancestry coefficient and kinship as covariates), and its improvements and modifications. To detect more MTAs with the least type I errors, multi-locus models have been proposed, which control background noise generated by other loci (termed pseudo-QTNs) which are in LD with the locus being tested. This is similar to the CIM and MIM strategies of QTL mapping. The multi-locus methods include MLMM (Segura et al., 2012), FarmCPU (Liu et al., 2016), and BLINK (Huang et al., 2019). Several other models were proposed after these recently (Zhang et al., 2020). Among them, in the present study, we have utilized these three models inbuilt in the GAPIT package for association analysis—MLM, FarmCPU, and BLINK (Lipka et al., 2012). In a GWAS study, it is important to keep the critical value very stringent to identify true MTAs by eliminating any false positives. Although the Bonferroni threshold is commonly used, it is likely to exclude some of the real positives if the probability of such MTAs lies too close to the threshold. Therefore, it is common to relax the threshold to bring such MTAs into selection. In this study too, the cutoff was relaxed to 5.0, but only those MTAs consistently appearing under multiple models were considered.

Although GWAS had been frequently adopted for mapping in rice, there were only two previous reports of GWAS for RSHT in rice. A set of 20 previously reported SSR markers was validated in one study using 62 rice genotypes (Pradhan et al., 2016). In another study, GBS-based genotyping of 167 rice accessions was performed to identify MTAs for 20 traits (Lafarge et al., 2017) using a set of 13,160, including 6667 SNPs and 6593 DArT markers. The authors compared three strategies of arriving at MTAs, single-marker-based regression, haplotype-based GWAS, and Bayesian Lasso-based analysis, which were utilized for identifying the MTAs, as in the present study. GWAS was also utilized for identifying genes for RSHT in other plant species such as Arabidopsis (Bac-Molenaar et al., 2015), *Brassica napus* (Rahaman et al., 2018), wheat (Tadesse et al., 2019; Guo et al., 2020; Sharma et al., 2020), and field pea (Tafesse et al., 2020).

Both *per se* trait values and the stress tolerance index extracted from them were utilized to establish marker-trait associations. GWAS for stress tolerance indices have been carried out in wheat (Gahlaut et al., 2019), cotton (Baytar et al., 2018), and *Brassica* (Khanzada et al., 2020). Several MTAs were identified in the present study for grain yield and spikelet fertility. The number of MTAs identified through FarmCPU and BLINK was higher than MLM, indicating that multi-locus models are effective in identifying reliable MTAs. False negatives were found to be more in the MLM model, implying its medium power of QTL detection. MLM weakens the associations in an effort to control the inflation of *p*-values *via* the population structure, thus missing out many minor effect MTAs. These minor effect MTAs are particularly relevant for complex traits such as heat stress tolerance. MTAs identified through the remaining two models cannot be deemed false positives given the appreciable phenotypic variances (*R* ^2^ values) observed for these MTAs. Hence, either FarmCPU or BLINK can be employed for GWAS (Merrick et al., 2022). Only nine previously reported QTLs were identified through the present study, indicating that most of the MTAs were novel. Two QTNs for grain yield per plant, five QTNs for spikelet fertility, and two QTNs for STISF were found to co-localize with previously reported QTLs. QTN for grain yield per plant identified through FarmCPU *qHTGY10.1* was co-localized with *qhr3-1* reported for heat tolerance by Cao et al. (2003) and *qHTGY11.1* overlapped with *qADL09-11* identified by Tazib et al. (2015) for anther dehiscence length. The QTN, *qHTST11.1* (or *qSTISF11.1* for STISF) for spikelet fertility identified in the present study through MLM coincided with two previously reported QTLs, *qHTSF11*.2 (Ye et al., 2015a) for spikelet fertility and *qLD10*-11 (Tazib et al., 2015) for longitudinal dehiscence of anthers. Another QTN, *qHTSF4.1* (and *qSTISF4.1* for STISF), for spikelet fertility on chromosome 4 coincided with *SSPF4* (Xiao et al., 2011a) and *qPF4* (Xiao et al., 2011b) mapped for seed set percentage and pollen fertility under heat stress, respectively. Furthermore, *qHTSF7.1* identified through FarmCPU for spikelet fertility co-localized with *qAL10-7* reported for anther length (Tazib et al., 2015). The MTA *qHTST2.1*, identified through BLINK, coincided with *qtl_2.2* reported for absolute spikelet fertility (Jagadish et al., 2010).

The regions identified as significant MTAs were analyzed using the genome browser on the Rice Genome Annotation Project website in order to identify putative candidate genes. The majority of those genes identified for grain yield per plant are involved in stress signaling, such as the glucosylceramidase (GCD) gene, zinc ion binding protein, DUF1336 domain-containing protein, cytochrome P450, diacylglycerol kinase, Ser/Thr protein kinase, OsWAK116–OsWAK receptor-like cytoplasmic kinase OsWAK-RLCK, and so forth. A DUF1336 domain-containing protein was identified on chromosome 8. Several transcriptomic studies reported differential expression of DUF domain-containing proteins under heat stress at the anthesis stage in rice, indicating their possible role in heat stress responses (Endo et al., 2009; Zhang et al., 2012; González-Schain et al., 2016). The cytochrome P450 proteins (like the one found on chromosome 8) have varied roles in the biotic and abiotic stress responses of plants, particularly in the synthesis of secondary metabolites (Jun et al., 2015; Pandian et al., 2020). Their relevance under heat stress has already been demonstrated in rice (Endo et al., 2009; González-Schain et al., 2016; Wang et al., 2019), mustard (Rahaman et al., 2018), *Panicum virgatum* (Li et al., 2013), *Rhazya stricta* (Obaid et al., 2016), and *Panicum maximum* (Wedow et al., 2019). Four kinase-encoding genes were found—one on chromosome 8, two on chromosome 1, and one on chromosome 11. Protein kinases are central to abiotic stress signal transduction pathways. Among these, *OsWAK116*, receptor-like wall-associated kinase proteins can be presumed to be a transducer of heat stress signal between the cytoplasm and cell wall (Zhang S. et al., 2005).

Several genes identified for spikelet fertility are involved in protein chaperoning pathways central to plant heat stress responses. The proteins containing the tetratricopeptide repeat (TRP) motif (like the one found on chromosome 11) are, in general, involved in protein–protein interactions which unite into multi-protein complexes to assist plants in warding off external stresses (Paeng et al., 2020). An E3 ubiquitin ligase identified on chromosome 1 might be involved in the degradation of denatured proteins due to heat stress. Ubiquitin ligases are frequently implicated under heat stress in rice (Mittal et al., 2012; Zhang et al., 2012; González-Schain et al., 2016). Broad-complex Tramtrack and the Bric-a-brac (BTB) domain/POX virus and Zinc finger (POZ) (BTB/POX) domain-containing proteins (chromosome 6) are implicated in transcriptional regulation and protein degradation (He et al., 2019). These BPM proteins are reported to negatively regulate the degradation of DREB2A and contribute to plant thermo-tolerance (Morimoto et al., 2017). The U-box domain-containing proteins (chromosome 3), called PUBs (plant U-box proteins), are a part of the ubiquitin–proteasome system (UPS) involved in the targeted ubiquitination and degradation of proteins when exposed to various environmental stresses (Hur et al., 2012; Byun et al., 2017; Ryu et al., 2019).

Ankyrin (ANK) repeat-containing proteins (chromosome 2) are involved in various protein–protein interactions (Michaely and Bennett, 1992; Li et al., 2006) and protein chaperoning (Shen et al., 2010) with putative roles in pollen germination and pollen tube growth in lily (Huang et al., 2006) and rice (Huang et al., 2009). These ANK repeat proteins also showed differential expression under heat stress at the anthesis stage in rice, in line with the present finding (González-Schain et al., 2016). RING finger proteins (chromosome 2) are a family of zinc finger proteins, with the majority being U3 ubiquitin ligases (Ciechanover 1998), which stem from the RING domain. There are reports of association of RING finger proteins in heat stress responses in rice. For instance, both *Oryza sativa* heat- and cold-induced 1(*OsHCI1*) and *Oryza sativa* heat-induced RING finger protein 1(*OsHIRP1*) act as E3 ligases and positively regulate heat stress responses (Lim et al., 2013; Kim et al., 2019). The rice *OsRZFP34* gene and *HEAT TOLERANCE AT SEEDLING STAGE* (*OsHTAS*) gene (an E3 ligase) improve high-temperature tolerance (Hsu et al., 2014; Liu et al., 2016). Corroborating this, the candidate gene of thermo-tolerance 1 (TT1) QTL identified from *O. glaberrima* encodes the α2 subunit of the 26S proteasome (Li et al., 2015). Several other genes such as DDT domain-containing protein, ATROPGEF7/ROPGEF7, phytochrome C, OsTOP6A1–Topoisomerase 6 subunit A homolog 1, transcription elongation factor protein, C3HC4-type domain-containing protein, PPR repeat-containing protein, universal stress protein domain-containing protein, deoxyuridine 5-triphosphate nucleotidohydrolase and WD domain, and G-beta repeat domain-containing protein were also found in the regions associated with spikelet fertility with putative functions in plant abiotic stress responses. An *in silico* expression analysis of these genes revealed an interesting pattern. The majority of the genes identified for spikelet fertility showed upregulation in reproductive and grain tissues, while the genes identified for grain yield showed higher expression levels in vegetative organs. Furthermore, nearly 10 genes matched with two heat stress-related DEGs identified in the previous transcriptomics studies (Cai et al., 2020; Liu et al., 2020). However, the logarithm of foldchange (Log_2_FC) values of the genes are <2.5 except for LOC_Os01g04580, a Ser/Thr protein kinase which was significantly downregulated at 6 h of heat stress exposure. This is understandable since the genotypes (SDWG005 and MH101) used in these two reports were completely different from those in the current study.

Thus, in the present study, significant reductions in grain yield and spikelet fertility were observed among the rice genotypes characterized for RSHT. GWAS identified many novel MTAs, explaining high phenotypic variance for both these traits. There was a clear difference between the effects of alternate alleles, indicating their significance in governing RSHT in rice. The majority of the candidate genes identified around these MTAs were either directly or indirectly involved in heat stress and other abiotic stress responses, which are valuable candidates for marker-assisted selection for the improvement of heat stress tolerance at a reproductive stage after further validation in future. Some uncharacterized genes were also observed for both grain yield and spikelet fertility, whose function needs to be elucidated in future studies.

## Data Availability

The datasets presented in this study can be found in online repositories. The name of the repository and link to the data can be found below: ICAR; https://krishi.icar.gov.in/jspui/handle/123456789/31947.
