# Supplementary figures and images for: Genome-Wide Association Mapping Reveals Novel Putative Gene Candidates Governing Reproductive Stage Heat Stress Tolerance in Rice
Source: Front Genet. 2022 May 10;13:876522. doi: 10.3389/fgene.2022.876522 (PMC9208292; doi:10.3389/fgene.2022.876522)

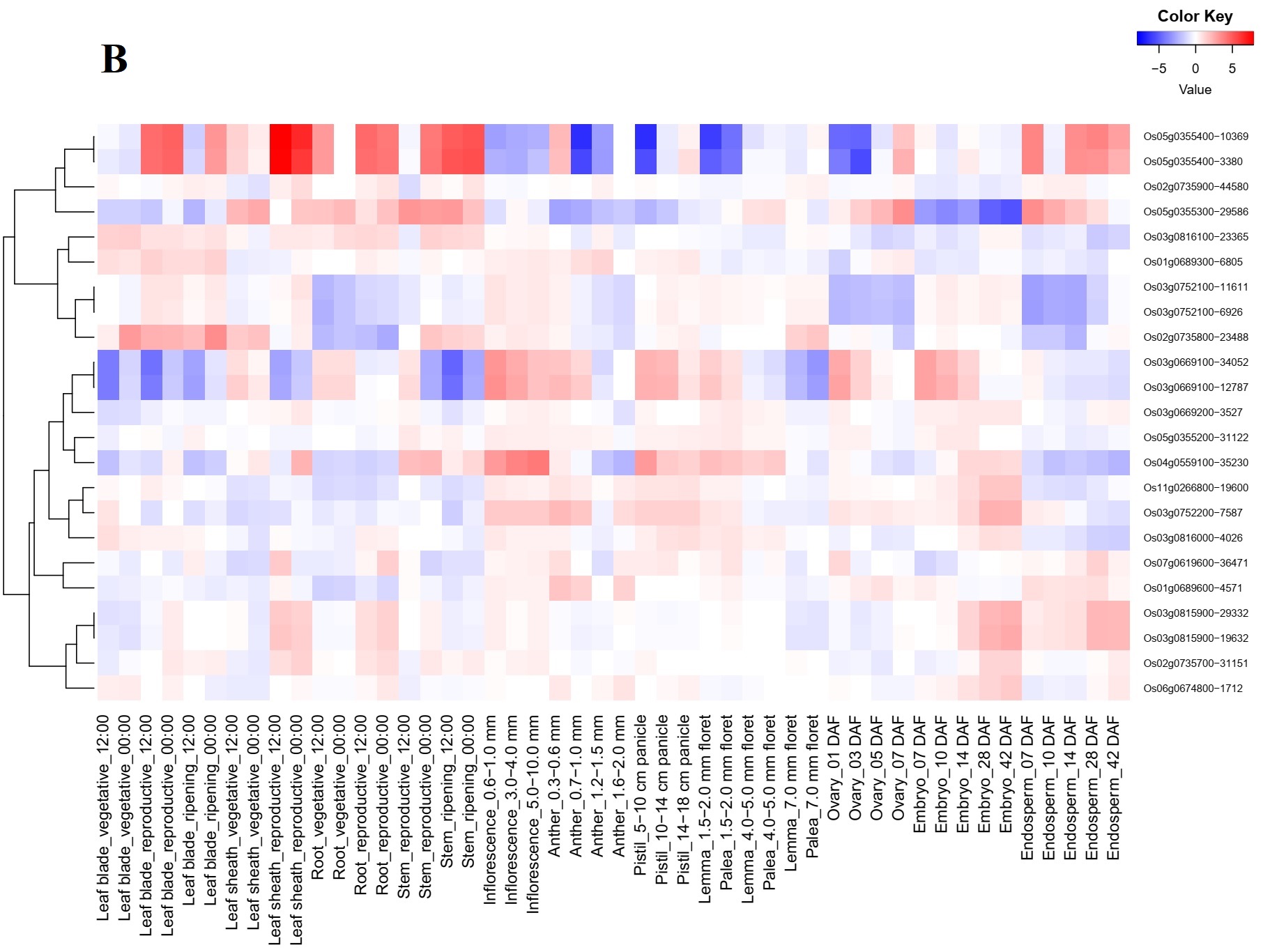

Supplement: Supplementary file 1 [file Image3.JPEG]

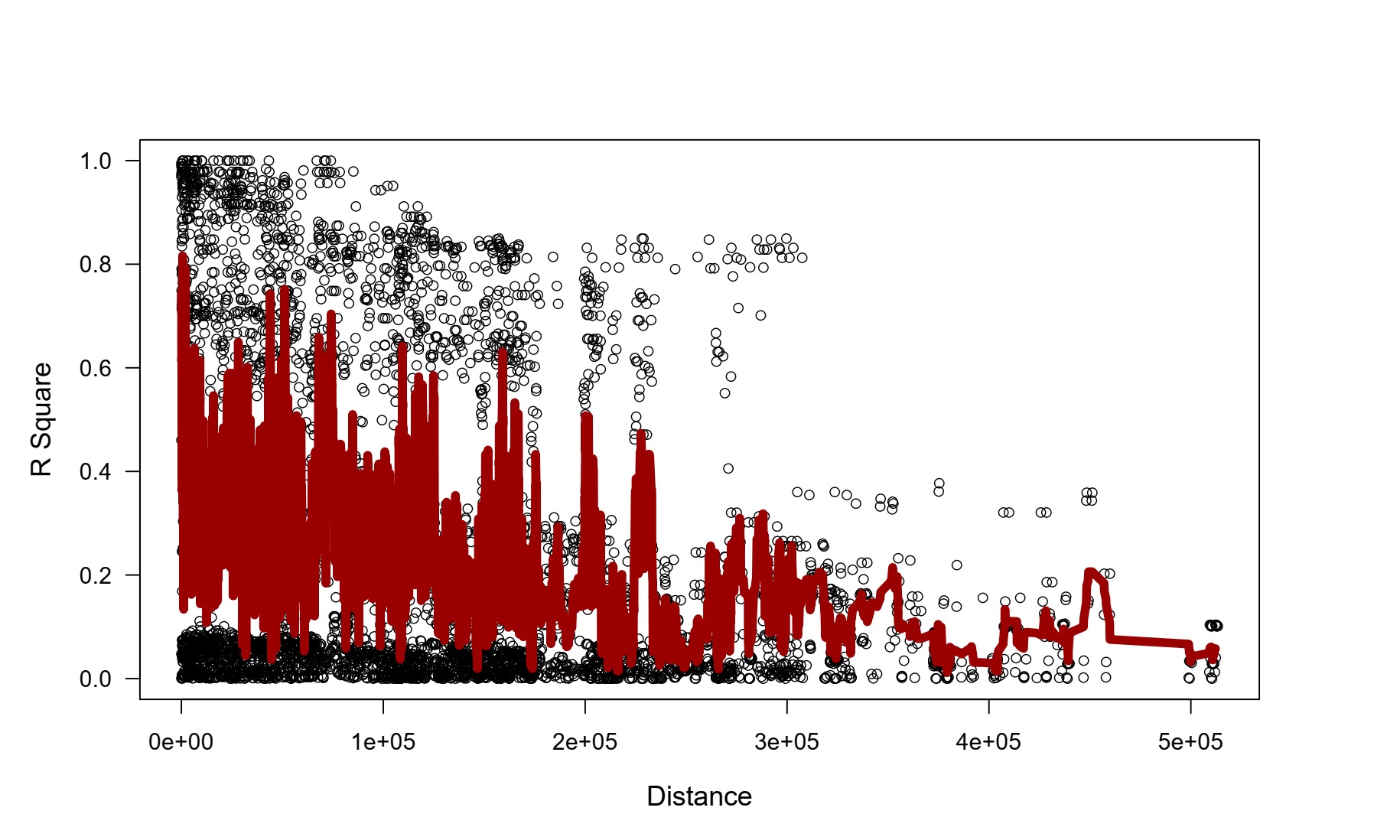

Supplement: Supplementary file 2 [file Image1.JPEG]

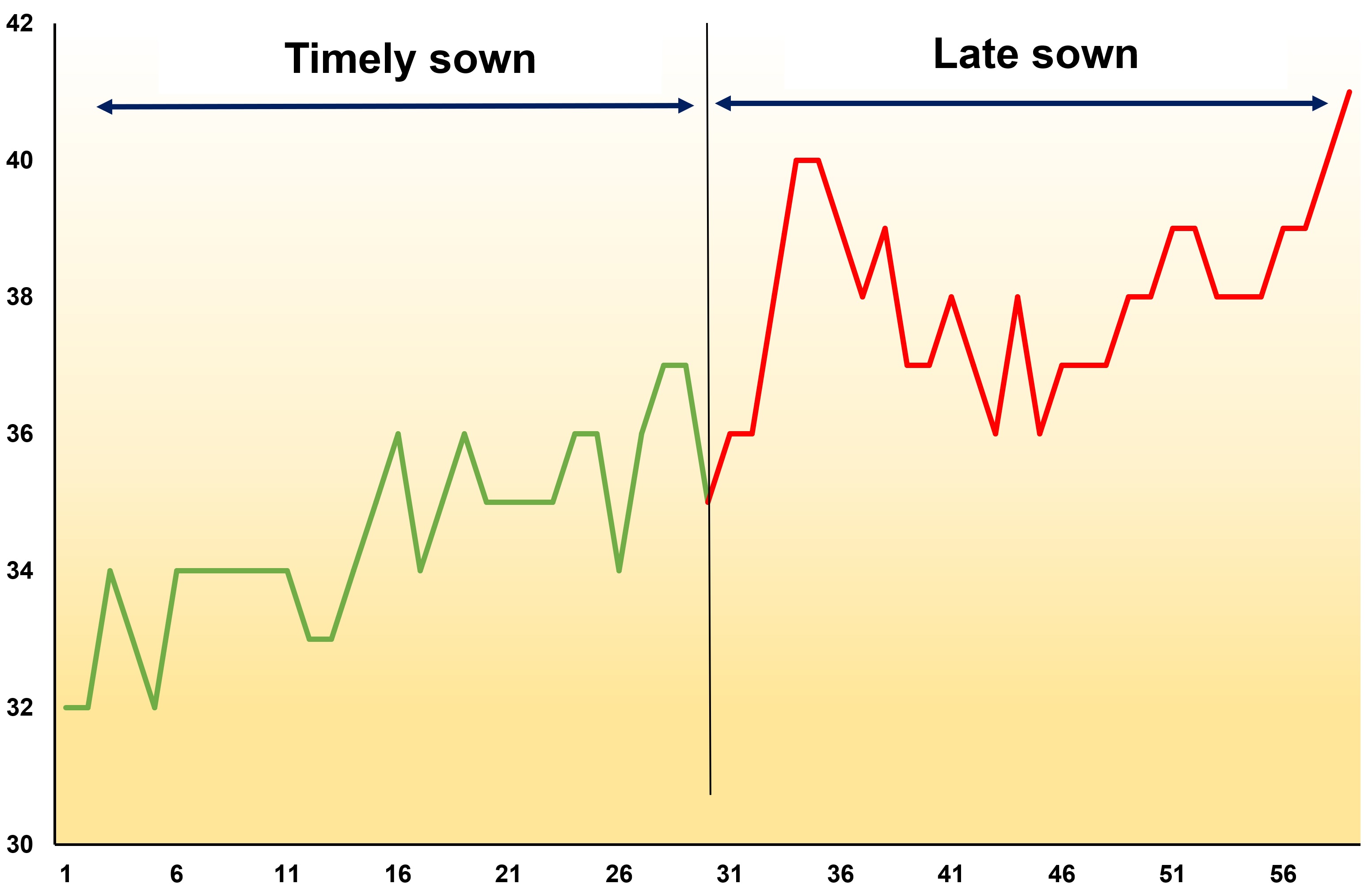

Supplement: Supplementary file 3 [file Image4.JPEG]

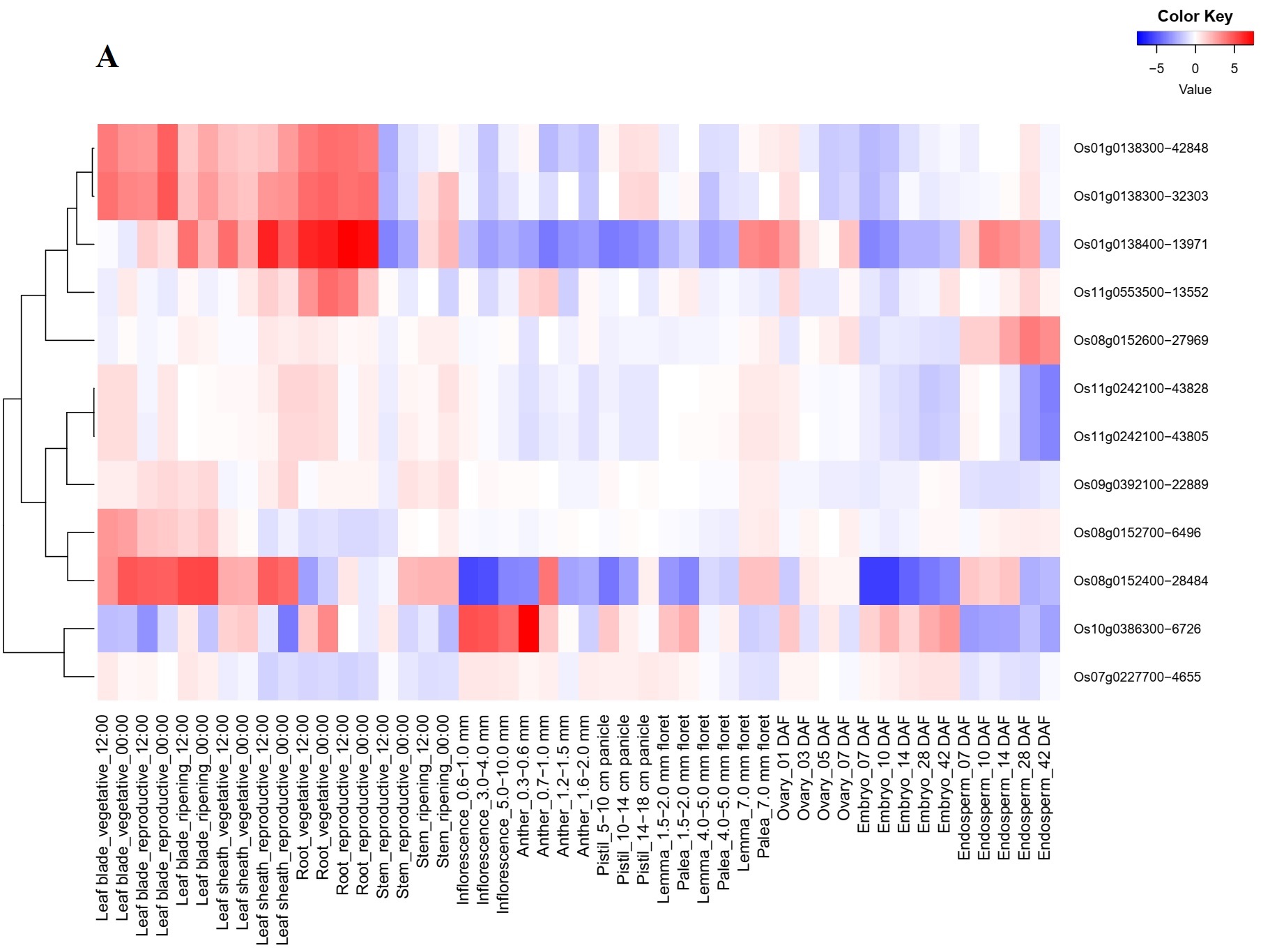

Supplement: Supplementary file 4 [file Image2.JPEG]
