# Supplementary material for: Genome-Wide Association Mapping Reveals Novel Putative Gene Candidates Governing Reproductive Stage Heat Stress Tolerance in Rice
Source: Front Genet. 2022 May 10;13:876522. doi: 10.3389/fgene.2022.876522 (PMC9208292; doi:10.3389/fgene.2022.876522)
Supplement: Supplementary file 5 [file DataSheet1.docx]

**Supplementary table 1**. Augmented RCB analysis of variance for grain yield and spikelet fertility under control and heat stress.

| Source of variation | Df | Grain yield under control | Grain yield under heat stress | Spikelet fertility under control | Spikelet fertility under heat stress |
| --- | --- | --- | --- | --- | --- |
| Treatment (ignoring Blocks) | 196 | 31.86** | 42.17** | 93.54** | 276.1** |
| Checks | 4 | 1.19* | 13.27* | 93.47 | 161.8** |
| Genotypes | 191 | 32.64** | 42.86** | 93.58* | 276.3** |
| Checks vs Genotypes | 1 | 6.14* | 24.69** | 84.36* | 689.1** |
| Blocks (eliminating treatments) | 3 | 5.27* | 22.16* | 111.93 | 150.4* |
| Residuals | 12 | 6.08 | 24.50 | 26.96 | 243.4 |

*Df, Degrees of freedom*

**Supplementary table 2**. Adjusted means of grain yield and spikelet fertility and their corresponding indices of 192 genotypes (along with checks) evaluated for reproductive heat stress tolerance during off-season, 2019 at Aduthurai, Tamil Nadu^$^.

| **S. No.** | **Genotype** | **Grain yield plant^-1^** | | | **Spikelet fertility** | | |
| --- | --- | --- | --- | --- | --- | --- | --- |
|  |  | Control | Heat stress | STI | Control | Heat stress | STI |
| 1 | CR 2461-9 | 19.50 | 17.80 | 0.83 | 91.32 | 67.35 | 0.90 |
| 2 | UPRI 2003-45 | 23.32 | 21.50 | 1.20 | 90.86 | 83.32 | 1.11 |
| 3 | PNR 381 | 25.55 | 16.75 | 1.02 | 87.21 | 67.32 | 0.86 |
| 4 | Punjab Mehak 1 | 21.41 | 15.80 | 0.81 | 86.25 | 63.14 | 0.80 |
| 5 | PNR 381 | 35.49 | 23.17 | 1.96 | 77.08 | 71.25 | 0.81 |
| 6 | Jayati | 33.54 | 18.25 | 1.46 | 80.21 | 76.76 | 0.90 |
| 7 | CT 10006-7-2M-5-1 P3-M | 30.37 | 9.67 | 0.70 | 72.63 | 55.19 | 0.59 |
| 8 | Chandrahasini | 38.13 | 14.63 | 1.33 | 80.91 | 75.80 | 0.90 |
| 9 | HPR 2143 | 17.82 | 13.25 | 0.56 | 83.16 | 43.32 | 0.53 |
| 10 | MAS 946-1 | 23.45 | 17.67 | 0.99 | 87.96 | 65.25 | 0.84 |
| 11 | PRR 126 | 17.00 | 15.18 | 0.62 | 83.33 | 72.03 | 0.88 |
| 12 | Ajay | 36.18 | 24.88 | 2.15 | 90.38 | 73.98 | 0.98 |
| 13 | RNRM 7 | 24.62 | 20.25 | 1.19 | 90.63 | 84.11 | 1.12 |
| 14 | Falguna | 22.18 | 19.38 | 1.03 | 85.89 | 70.03 | 0.88 |
| 15 | VLT-6 | 18.85 | 18.67 | 0.84 | 85.82 | 77.01 | 0.97 |
| 16 | RIL 10 | 29.87 | 23.50 | 1.68 | 90.59 | 86.23 | 1.14 |
| 17 | Pant dhan 19 | 33.05 | 23.50 | 1.85 | 83.00 | 67.78 | 0.82 |
| 18 | NDR 8015-1 | 23.68 | 22.00 | 1.24 | 80.61 | 69.99 | 0.83 |
| 19 | Pusa Sugandh 3 | 25.08 | 15.40 | 0.92 | 85.52 | 76.29 | 0.96 |
| 20 | Bala | 9.77 | 9.00 | 0.21 | 79.30 | 68.65 | 0.80 |
| 21 | Pant sankar dhan 3 | 19.39 | 16.30 | 0.75 | 80.47 | 70.00 | 0.83 |
| 22 | Samanta | 26.00 | 24.90 | 1.55 | 84.39 | 74.98 | 0.93 |
| 23 | Tapaswani | 25.82 | 19.13 | 1.18 | 86.37 | 69.75 | 0.88 |
| 24 | HPR 2083 | 24.57 | 22.50 | 1.32 | 76.02 | 34.30 | 0.38 |
| 25 | Birupa | 20.88 | 19.87 | 0.99 | 87.72 | 74.62 | 0.96 |
| 26 | Bhuban | 30.83 | 26.24 | 1.93 | 94.70 | 75.35 | 1.05 |
| 27 | Pant dhan 10 | 21.09 | 18.33 | 0.92 | 92.22 | 75.78 | 1.02 |
| 28 | OYR 128 | 23.25 | 18.99 | 1.05 | 82.82 | 79.84 | 0.97 |
| 29 | OYC 183 | 20.31 | 19.80 | 0.96 | 94.02 | 80.22 | 1.11 |
| 30 | BJ 1 | 16.50 | 14.73 | 0.58 | 88.96 | 66.67 | 0.82 |
| 31 | Chandana | 11.59 | 9.50 | 0.26 | 79.74 | 33.83 | 0.40 |
| 32 | Urvashi | 15.46 | 14.10 | 0.52 | 88.78 | 61.12 | 0.80 |
| 33 | Pant dhan 16 | 18.17 | 17.73 | 0.77 | 91.94 | 53.91 | 0.73 |
| 34 | P 1490-03 | 15.02 | 7.00 | 0.25 | 48.74 | 27.12 | 0.19 |
| 35 | CSR 27 | 24.10 | 16.80 | 0.97 | 93.10 | 76.13 | 1.04 |
| 36 | China 988 | 13.83 | 10.14 | 0.33 | 85.26 | 77.02 | 0.96 |
| 37 | DV 85 | 25.47 | 17.13 | 1.04 | 94.09 | 91.46 | 1.26 |
| 38 | IRAT 240 | 25.91 | 18.33 | 1.13 | 89.30 | 83.47 | 1.09 |
| 39 | CR 2363-26 | 13.75 | 7.38 | 0.24 | 73.88 | 44.34 | 0.48 |
| 40 | Selected Sabarmati | 21.98 | 20.38 | 1.07 | 86.80 | 86.79 | 1.10 |
| 41 | MTU 1010 | 25.83 | 20.60 | 1.27 | 95.07 | 78.79 | 1.10 |
| 42 | Kataktara | 19.61 | 16.50 | 0.77 | 75.59 | 74.45 | 0.82 |
| 43 | VOH-PCR-3110 | 23.98 | 19.30 | 1.11 | 84.05 | 76.13 | 0.94 |
| 44 | Karupunel | 16.50 | 12.75 | 0.50 | 88.25 | 75.76 | 0.98 |
| 45 | Kasturi | 15.33 | 11.41 | 0.42 | 81.98 | 80.67 | 0.97 |
| 46 | C 1268-7-10 | 23.42 | 20.63 | 1.15 | 94.23 | 54.01 | 0.75 |
| 47 | BJ 1 | 26.49 | 16.75 | 1.06 | 90.54 | 90.18 | 1.20 |
| 48 | VL 88-97-1-7 | 18.09 | 14.00 | 0.60 | 77.41 | 75.75 | 0.86 |
| 49 | Kamlesh | 15.47 | 13.70 | 0.51 | 92.04 | 87.71 | 1.18 |
| 50 | IR 78908 | 15.20 | 8.44 | 0.31 | 76.93 | 69.84 | 0.79 |
| 51 | B6144-MR-6-0-0 | 16.81 | 15.33 | 0.62 | 83.59 | 82.60 | 1.01 |
| 52 | Seond Basmati | 6.98 | 6.43 | 0.11 | 52.36 | 28.11 | 0.22 |
| 53 | HPR 2104 | 12.45 | 10.74 | 0.32 | 68.12 | 46.26 | 0.46 |
| 54 | Shah Pasand | 10.09 | 9.70 | 0.23 | 56.01 | 31.77 | 0.26 |
| 55 | Chimbalate Basmati | 20.78 | 19.71 | 0.98 | 85.68 | 68.16 | 0.86 |
| 56 | PMK 1 | 29.74 | 21.14 | 1.50 | 78.18 | 64.00 | 0.73 |
| 57 | Sitwa Dhan | 26.15 | 24.66 | 1.54 | 77.49 | 75.02 | 0.85 |
| 58 | Gouri | 27.44 | 24.90 | 1.63 | 78.06 | 76.81 | 0.88 |
| 59 | Ananga | 17.62 | 13.61 | 0.57 | 87.73 | 61.24 | 0.79 |
| 60 | Pant dhan 4 | 20.10 | 17.90 | 0.86 | 86.27 | 71.30 | 0.90 |
| 61 | OYR 69 | 8.49 | 2.14 | 0.04 | 55.61 | 43.53 | 0.35 |
| 62 | Khuch | 8.85 | 8.65 | 0.18 | 75.83 | 60.96 | 0.68 |
| 63 | CR 2499 | 26.90 | 18.48 | 1.19 | 91.97 | 87.03 | 1.17 |
| 64 | Pusa 1460 | 21.26 | 17.69 | 0.90 | 79.33 | 69.66 | 0.81 |
| 65 | Local collection | 22.09 | 19.20 | 0.81 | 77.51 | 66.98 | 0.82 |
| 66 | UPRI 2003-24 | 24.36 | 17.71 | 1.03 | 90.79 | 77.41 | 1.03 |
| 67 | Poornima | 12.42 | 11.67 | 0.35 | 67.27 | 61.84 | 0.61 |
| 68 | UPRI 2003-18 | 20.14 | 19.96 | 0.96 | 89.27 | 79.68 | 1.04 |
| 69 | Narendra Usar Dhan III | 25.23 | 21.54 | 1.30 | 91.96 | 79.78 | 1.08 |
| 70 | NDR 359 | 32.02 | 23.49 | 1.80 | 90.35 | 82.57 | 0.82 |
| 71 | NDR 97 | 27.22 | 16.85 | 1.10 | 90.99 | 82.95 | 1.11 |
| 72 | Bhadrakali | 25.50 | 21.54 | 1.31 | 90.73 | 89.81 | 1.19 |
| 73 | Shiva | 29.98 | 10.27 | 0.74 | 85.34 | 73.49 | 0.92 |
| 74 | IR 77384-12-35-3-6-7-2-B | 17.23 | 15.20 | 0.81 | 87.58 | 83.88 | 1.08 |
| 75 | Narendra Usar Dhan II | 28.73 | 22.19 | 1.52 | 74.15 | 73.68 | 0.80 |
| 76 | SKAU 220 | 12.59 | 8.54 | 0.26 | 85.52 | 73.77 | 0.92 |
| 77 | PRR 105 | 19.33 | 18.00 | 0.83 | 68.15 | 59.23 | 0.59 |
| 78 | PRR 117 | 16.74 | 14.53 | 0.58 | 87.13 | 85.58 | 1.09 |
| 79 | PRR 103 | 21.12 | 18.60 | 0.94 | 81.18 | 68.34 | 0.81 |
| 80 | PRR 123 | 21.49 | 17.74 | 0.91 | 79.47 | 68.08 | 0.79 |
| 81 | PRR 114 | 20.80 | 14.66 | 0.73 | 91.28 | 73.37 | 0.98 |
| 82 | PRR 120 | 17.55 | 15.18 | 0.64 | 78.89 | 78.58 | 0.91 |
| 83 | PRR 115 | 18.57 | 17.37 | 0.77 | 86.78 | 71.02 | 0.90 |
| 84 | PRR 104 | 23.11 | 17.01 | 0.94 | 89.91 | 73.76 | 0.97 |
| 85 | PRR 108 | 21.63 | 12.89 | 0.67 | 80.18 | 62.52 | 0.73 |
| 86 | PRR 121 | 20.84 | 19.26 | 0.96 | 87.74 | 74.77 | 0.96 |
| 87 | Pant dhan 18 | 19.98 | 19.27 | 0.92 | 91.15 | 88.72 | 1.19 |
| 88 | Indravati | 27.88 | 25.56 | 1.70 | 83.64 | 82.81 | 1.02 |
| 89 | Pant Sugandh Dhan 17 | 16.11 | 14.30 | 0.55 | 86.46 | 70.71 | 0.90 |
| 90 | HUR 200-57-1 | 23.37 | 11.11 | 0.62 | 85.75 | 53.93 | 0.68 |
| 91 | Pant dhan 15 | 26.08 | 5.54 | 0.35 | 81.76 | 25.54 | 0.31 |
| 92 | JGL 11727 | 23.64 | 15.37 | 0.87 | 83.65 | 73.67 | 0.90 |
| 93 | Mahanadi | 33.65 | 14.21 | 1.14 | 80.29 | 63.08 | 0.74 |
| 94 | Pant Sugandh Dhan15 | 18.06 | 6.64 | 0.29 | 79.95 | 23.65 | 0.28 |
| 95 | Bhuman San | 21.49 | 7.59 | 0.39 | 84.51 | 35.71 | 0.44 |
| 96 | JR 75 | 23.58 | 19.88 | 1.12 | 93.18 | 87.06 | 1.19 |
| 97 | CO 37 | 14.12 | 12.25 | 0.41 | 72.13 | 71.11 | 0.75 |
| 98 | Sumati | 18.45 | 6.83 | 0.30 | 78.21 | 19.17 | 0.22 |
| 99 | SAF 1221-83 | 8.68 | 7.34 | 0.15 | 75.87 | 62.49 | 0.70 |
| 100 | P 1447 | 17.94 | 7.48 | 0.32 | 76.82 | 65.17 | 0.73 |
| 101 | P 1447-00-5-1 | 19.61 | 15.22 | 0.71 | 87.00 | 67.87 | 0.87 |
| 102 | HUR 105 | 35.42 | 24.42 | 2.07 | 87.48 | 65.65 | 0.84 |
| 103 | Manaswini | 16.36 | 13.23 | 0.52 | 67.07 | 61.19 | 0.60 |
| 104 | RR 8585 | 21.97 | 21.90 | 1.15 | 88.14 | 76.62 | 0.99 |
| 105 | CSR 23 | 29.13 | 20.66 | 1.44 | 79.93 | 71.71 | 0.84 |
| 106 | Kudrat-3 | 22.37 | 12.31 | 0.66 | 93.41 | 84.01 | 1.15 |
| 107 | RAU 3061 | 28.67 | 21.23 | 1.45 | 82.57 | 65.72 | 0.82 |
| 108 | NDR 625 | 23.60 | 14.07 | 0.79 | 80.10 | 60.98 | 0.72 |
| 109 | Sagar Dambha | 15.15 | 14.18 | 0.51 | 71.88 | 64.27 | 0.68 |
| 110 | JGL 3828 | 23.45 | 18.45 | 1.03 | 94.23 | 82.76 | 1.14 |
| 111 | P 1460 | 23.64 | 11.57 | 0.65 | 85.71 | 69.62 | 0.87 |
| 112 | Muskan | 23.97 | 19.28 | 1.10 | 90.47 | 76.29 | 1.01 |
| 113 | Pusa 1342 | 11.24 | 6.66 | 0.18 | 66.85 | 50.82 | 0.50 |
| 114 | MR 219 | 23.99 | 17.17 | 0.98 | 90.80 | 74.71 | 0.99 |
| 115 | UPRI 2003-15 | 20.01 | 19.73 | 0.94 | 91.49 | 79.40 | 1.06 |
| 116 | Sambha Mahsuri | 24.23 | 22.32 | 1.29 | 88.64 | 78.24 | 1.02 |
| 117 | Sharbati | 26.69 | 16.75 | 1.07 | 80.39 | 74.13 | 0.87 |
| 118 | HKR 200-57-1 | 22.39 | 20.23 | 1.08 | 93.44 | 73.28 | 1.00 |
| 119 | WGL 23985 | 23.37 | 18.12 | 1.01 | 84.05 | 75.97 | 0.94 |
| 120 | Rajendra basmati | 24.99 | 14.25 | 0.85 | 82.57 | 60.92 | 0.82 |
| 121 | MR 220 | 24.13 | 23.09 | 1.33 | 78.77 | 69.27 | 0.80 |
| 122 | Raja Vaellu | 24.01 | 15.52 | 0.89 | 78.95 | 69.00 | 0.80 |
| 123 | CR 246-16 | 20.19 | 17.28 | 0.83 | 81.04 | 73.87 | 0.88 |
| 124 | Phunchi | 19.07 | 10.45 | 0.48 | 80.81 | 70.48 | 0.83 |
| 125 | Pusa Basmati 1121 | 19.48 | 8.68 | 0.40 | 58.13 | 51.76 | 0.44 |
| 126 | Improved Sabarmati | 26.30 | 16.27 | 1.02 | 78.92 | 68.63 | 0.79 |
| 127 | Tompha Khau | 9.06 | 8.44 | 0.18 | 82.36 | 74.34 | 0.90 |
| 128 | CN-1268-7 | 18.08 | 16.83 | 0.73 | 84.24 | 62.17 | 0.77 |
| 129 | WGL 14 | 30.34 | 19.61 | 1.42 | 78.58 | 72.43 | 0.83 |
| 130 | Hema | 24.88 | 22.22 | 1.32 | 86.79 | 86.01 | 1.09 |
| 131 | UPRVS 8-26 | 20.47 | 18.36 | 0.81 | 82.57 | 73.58 | 0.82 |
| 132 | Super Basmati | 17.37 | 12.16 | 0.50 | 75.29 | 62.85 | 0.69 |
| 133 | Pusa 1301 | 10.30 | 5.32 | 0.13 | 91.57 | 40.34 | 0.54 |
| 134 | Kanak | 18.38 | 14.96 | 0.66 | 84.55 | 79.72 | 0.99 |
| 135 | PRR 109 | 18.88 | 15.95 | 0.72 | 70.94 | 66.67 | 0.82 |
| 136 | PRR 110 | 19.03 | 12.78 | 0.58 | 58.44 | 46.24 | 0.40 |
| 137 | PRR 127 | 31.57 | 25.42 | 1.92 | 89.96 | 71.46 | 0.94 |
| 138 | PRR 122 | 26.20 | 24.93 | 1.56 | 91.97 | 88.52 | 1.19 |
| 139 | PRR 118 | 6.53 | 3.06 | 0.05 | 33.85 | 5.30 | 0.03 |
| 140 | HUR 36 | 19.85 | 17.64 | 0.84 | 89.59 | 54.77 | 0.72 |
| 141 | RR 166-645 | 7.23 | 6.23 | 0.11 | 52.70 | 51.21 | 0.40 |
| 142 | Nirri | 25.89 | 20.46 | 1.26 | 74.49 | 63.99 | 0.70 |
| 143 | C 22 | 21.35 | 19.27 | 0.98 | 92.75 | 73.71 | 1.00 |
| 144 | DHMAS-70G-164-29 | 16.74 | 5.60 | 0.22 | 75.46 | 53.36 | 0.59 |
| 145 | Ranbir Basmati | 18.73 | 11.31 | 0.51 | 82.42 | 28.45 | 0.34 |
| 146 | T23 | 26.89 | 24.64 | 1.58 | 87.92 | 75.62 | 0.97 |
| 147 | Langphou | 22.79 | 15.03 | 0.82 | 77.55 | 61.81 | 0.70 |
| 148 | ASD 19 | 25.35 | 20.20 | 1.22 | 91.05 | 45.39 | 0.61 |
| 149 | Basmati 370 | 15.78 | 13.39 | 0.50 | 80.98 | 70.83 | 0.84 |
| 150 | BPT 5204 | 18.87 | 13.26 | 0.60 | 82.31 | 69.28 | 0.84 |
| 151 | IC 2127 | 22.84 | 15.11 | 0.82 | 88.91 | 79.89 | 1.04 |
| 152 | VOH-PCR-3113 | 18.53 | 16.62 | 0.74 | 82.49 | 67.39 | 0.81 |
| 153 | TYPE 3 | 16.04 | 13.86 | 0.53 | 90.40 | 78.40 | 1.04 |
| 154 | Nagina 12 | 22.61 | 17.13 | 0.92 | 82.64 | 70.68 | 0.86 |
| 155 | Sonasal | 16.81 | 8.88 | 0.36 | 71.20 | 65.44 | 0.68 |
| 156 | Khara Munga | 14.76 | 11.65 | 0.41 | 91.99 | 74.15 | 1.00 |
| 157 | IR 70 | 25.04 | 22.48 | 1.34 | 81.31 | 80.29 | 0.96 |
| 158 | ADT 39 | 29.21 | 23.57 | 1.64 | 89.85 | 79.38 | 1.05 |
| 159 | Haldimuri | 25.14 | 23.38 | 1.40 | 97.52 | 88.35 | 1.26 |
| 160 | MTU 7029 | 23.77 | 21.75 | 1.23 | 91.73 | 79.82 | 1.07 |
| 161 | PDKV Chinoor 2 | 21.62 | 7.71 | 0.40 | 81.73 | 65.37 | 0.78 |
| 162 | Swarna Sub 1 | 21.96 | 17.16 | 0.90 | 79.67 | 71.31 | 0.83 |
| 163 | Tilak Chandan | 11.67 | 8.08 | 0.23 | 86.69 | 53.46 | 0.68 |
| 164 | Pusa 33 | 22.95 | 19.65 | 1.08 | 76.91 | 75.74 | 0.85 |
| 165 | Pratikshya | 22.85 | 17.57 | 0.96 | 92.26 | 74.31 | 1.00 |
| 166 | Jhulhat | 21.16 | 11.74 | 0.59 | 82.57 | 72.42 | 0.82 |
| 167 | NDR 9830144 | 20.33 | 13.88 | 0.67 | 89.83 | 76.62 | 1.01 |
| 168 | MTU 1001 | 29.12 | 17.97 | 1.25 | 90.40 | 83.06 | 1.10 |
| 169 | Bamleshwari | 30.65 | 11.25 | 0.82 | 82.57 | 83.28 | 0.82 |
| 170 | Ramachandi | 24.27 | 18.67 | 1.08 | 91.43 | 86.57 | 1.16 |
| 171 | Local collection | 20.35 | 8.31 | 0.40 | 88.08 | 75.20 | 0.97 |
| 172 | Apo | 17.30 | 16.03 | 0.66 | 91.41 | 73.60 | 0.99 |
| 173 | Aziz Beoul | 11.76 | 7.88 | 0.22 | 82.80 | 67.42 | 0.82 |
| 174 | Begum | 9.17 | 3.17 | 0.07 | 83.52 | 10.26 | 0.45 |
| 175 | Bala Koun | 6.82 | 5.49 | 0.09 | 57.28 | 53.37 | 0.45 |
| 176 | Buta Baber | 7.24 | 5.00 | 0.09 | 81.28 | 57.67 | 0.69 |
| 177 | Baber | 13.16 | 5.09 | 0.16 | 87.13 | 57.09 | 0.73 |
| 178 | Baber Safed | 12.52 | 9.18 | 0.27 | 84.61 | 34.75 | 0.43 |
| 179 | Budgi | 13.15 | 4.99 | 0.16 | 83.74 | 21.88 | 0.27 |
| 180 | Gull Baber | 10.75 | 8.80 | 0.23 | 64.00 | 46.46 | 0.44 |
| 181 | Kaw Qudder | 8.23 | 8.03 | 0.16 | 73.53 | 54.58 | 0.59 |
| 182 | Kew | 8.65 | 7.00 | 0.14 | 62.22 | 35.16 | 0.32 |
| 183 | Mehvan (purple) | 10.67 | 5.29 | 0.13 | 75.58 | 33.43 | 0.37 |
| 184 | Mehvan (green) | 9.72 | 6.82 | 0.16 | 83.19 | 31.98 | 0.39 |
| 185 | Mir Zug | 4.47 | 1.04 | 0.01 | 77.98 | 44.86 | 0.51 |
| 186 | CO 50 | 14.79 | 6.71 | 0.24 | 90.25 | 45.03 | 0.60 |
| 187 | CO 51 | 16.11 | 15.41 | 0.59 | 95.95 | 83.46 | 1.17 |
| 188 | Arupathaam Kuruvai | 19.35 | 12.53 | 0.58 | 79.16 | 70.58 | 0.82 |
| 189 | Improved Samba Mahsuri | 17.08 | 16.84 | 0.69 | 96.54 | 90.43 | 1.28 |
| 190 | Sabour Surbhit | 17.70 | 13.56 | 0.57 | 70.13 | 66.51 | 0.68 |
| 191 | North Andaman 2 | 17.58 | 15.87 | 0.67 | 94.92 | 89.38 | 1.24 |
| 192 | Local collection | 10.73 | 5.82 | 0.15 | 84.73 | 61.57 | 0.76 |
| C1 | NL44 | 25.21 | 20.94 | 1.24 | 90.53 | 87.92 | 1.16 |
| C2 | Pusa Basmati 1 | 23.04 | 13.76 | 0.75 | 88.57 | 56.85 | 0.74 |
| C3 | N22 | 21.89 | 20.16 | 1.04 | 96.21 | 95.87 | 1.34 |
| C4 | IR64 | 29.72 | 18.27 | 1.28 | 87.51 | 75.96 | 0.97 |
| C5 | PB1509 | 25.57 | 12.15 | 0.73 | 86.24 | 49.89 | 0.63 |
| C.D. (5%) _Checks_ | | 3.50 | 2.74 | - | 3.67 | 11.03 | - |
| C.D. (5%) _Genotypes of same block_ | | 7.00 | 3.49 | - | 7.34 | 22.06 | - |
| C.D. (5%) _Genotypes of different blocks_ | | 7.67 | 3.82 | - | 8.04 | 24.17 | - |
| C.D. (5%) _Checks vs test genotypes_ | | 5.86 | 2.92 | - | 6.14 | 18.46 | - |

**^$^**The mean of checks across blocks were given
